# Pterostilbene inhibits non-small cell lung cancer progression by activating the STING pathway and enhancing antitumor immune response

**DOI:** 10.3389/fimmu.2025.1622284

**Published:** 2025-10-16

**Authors:** Li-Ping Kang, Han Xie, Hua-Jing Huang, Pan Xu, Cong Xu, Dong-Hui Huang, Ze-Bo Jiang

**Affiliations:** ^1^ Zhuhai Hospital of Integrated Traditional Chinese and Western Medicine, Zhuhai, Guangdong, China; ^2^ Department of Traditional Chinese Medicine, The Third Affiliated Hospital of Sun Yat-sen University, Guangzhou, China; ^3^ Northeastern University, Boston, MA, United States; ^4^ Department of Oncology, The Affiliated Cancer Hospital of Nanjing Medical University and Jiangsu Cancer Hospital and Jiangsu Institute of Cancer Research, Nanjing, China

**Keywords:** pterostilbene, non-small cell lung cancer, STING pathway, reactive oxygen species, CD8+ T, tumor microenvironment

## Abstract

**Introduction:**

Non-small cell lung cancer (NSCLC) is a leading cause of cancer-related mortality, and current therapies often yield limited efficacy. This study investigated the antitumor potential and mechanisms of Pterostilbene (PTE), a natural stilbenoid with superior bioavailability.

**Methods:**

The antitumor effects of PTE were assessed in A549 and H358 NSCLC cell lines to determine its impact on cell viability, cell cycle, apoptosis, and reactive oxygen species (ROS) generation, using N-acetylcysteine (NAC) to confirm the role of ROS. Key molecular mechanisms were probed via Western blot, siRNA knockdown, and pharmacological inhibition (H-151). The in vivo efficacy of PTE and its effect on the tumor immune microenvironment were evaluated in H358 xenograft and immunocompetent LLC1 murine models.

**Results:**

PTE suppressed cell viability in a concentration- and time-dependent manner, inducing G2/M phase arrest and mitochondrial apoptosis driven by ROS. It triggered DNA damage and activated the STING pathway, leading to TBK1/IRF3 phosphorylation and the secretion of T-cell chemoattractants (CXCL10, CXCL9, CCL5). STING inhibition markedly attenuated PTE's effects. *In vivo*, PTE suppressed tumor growth and remodeled the tumor microenvironment by increasing granzyme B^+^, TNF-α^+^, and IFN-γ^+^ CD8^+^ T cells while reducing myeloid-derived suppressor cells and regulatory T cells.

**Discussion:**

Our findings elucidate a dual mechanism whereby PTE directly kills NSCLC cells via ROS-mediated apoptosis and simultaneously reinvigorates antitumor immunity through STING pathway activation. This positions PTE as a promising candidate for combination immunotherapy in NSCLC.

## 1 Introduction

Lung cancer is the leading cause of cancer-related deaths globally, with approximately 2.5 million new cases and nearly 1.8 million deaths reported in 2022 (1, 2). Non-small cell lung cancer (NSCLC) constitutes the majority of cases and is frequently diagnosed at advanced stages, where therapeutic options remain limited and prognosis is poor (3). Current management strategies include surgical resection, radiotherapy, chemotherapy, molecularly targeted agents, and immunotherapy. However, the overall survival benefit from these interventions, particularly for metastatic disease, remains unsatisfactory. Chemotherapy, though widely used, is often associated with substantial toxicity and the emergence of drug resistance, underscoring the need for more precise and tolerable treatments (4, 5). Furthermore, intertumoral and intratumoral heterogeneity contribute to acquired resistance, ultimately diminishing therapeutic efficacy (6).

Immunotherapies, especially immune checkpoint inhibitors (ICIs) targeting the programmed cell death protein 1 (PD-1)/Programmed Cell Death Ligand 1 (PD-L1) axis, have revolutionized oncology and represents a promising modality for NSCLC (7). By blocking PD-1 on immune cells such as CD8^+^ T cells, dendritic cells (DCs), macrophages, and regulatory T cells (Tregs), ICIs reinvigorate antitumor immunity and have demonstrated durable clinical responses in a subset of patients (8, 9). Nonetheless, resistance to ICIs is common, prompting investigations into the immunosuppressive mechanisms within the tumor microenvironment (TME) that facilitate immune evasion (10). Infiltration and functional activation of CD8^+^ T cells in the TME are strongly correlated with favorable outcomes in NSCLC, making them a key focus for therapeutic enhancement (11, 12). Current strategies to amplify T cell-mediated immunity include combining ICIs with chemotherapy, immunomodulatory agents, or personalized cancer vaccines (13). However, immunosuppressive cell populations, such as Tregs, tumor-associated macrophages (TAMs), and myeloid-derived suppressor cells (MDSCs), often undermine these efforts by establishing an inhibitory niche (14–16). Targeting these cells has therefore emerged as a viable approach to augment immunotherapy (17).

Natural products and traditional Chinese medicine offer a rich source of bioactive compounds with anticancer potential. *Pterocarpus marsupium (PM) heartwood*, used in traditional medicine, contains the stilbenoid Pterostilbene (PTE), which has attracted scientific interest for its pleiotropic health benefits (18). PTE demonstrates antioxidative, anti-inflammatory, and antineoplastic properties across various malignancies, including lung, pancreatic, and gastric cancers (18–20). Recent studies suggest that PTE can overcome resistance to tyrosine kinase inhibitors (TKIs) and enhance sensitivity to radiation therapy in cancer patients (21, 22). However, its mechanisms of action against NSCLC remain largely unexplored. Traditional Chinese medicine has shown promise in enhancing tumor immunotherapy through compounds such as polysaccharides (23, 24). Nevertheless, the mechanisms underlying PTE’s antitumor activity in NSCLC-particularly those involving innate immune activation and TME remodeling-are not fully elucidated.

In this study, we investigated the effects of PTE on cell survival, division, and apoptosis in human NSCLC cell lines. Our findings indicated that PTE effectively inhibited the growth of NSCLC cells. Additionally, we observed that PTE activated the Stimulator of Interferon Response (STING)/TANK-binding kinase 1 (TBK1)/Interferon Regulatory Factor 3 (IRF3) pathway, resulting in upregulation of C-X-C Motif Chemokine Ligand 10 (CXCL10) and C-C Motif Chemokine Ligand 5 (CCL5), which may enhance the recruitment of immune cells, including macrophages, DCs, and CD8^+^ T cells, into the TME. Notably, PTE demonstrated significant antitumor effects and stimulated antitumor immunity in a murine LLC1 lung cancer model. These results highlight the potential therapeutic benefits of PTE in NSCLC treatment by promoting tumor cell death and modulating the immune response within the TME.

## 2 Materials and methods

### 2.1 Materials

In this study, pharmaceutical-grade PTE with a purity of at least 98% was obtained from Shanghai Yuanye Bio-Technology Co., Ltd (Shanghai, China). PTE was dissolved in dimethyl sulfoxide (DMSO) and stored at -20 °C until further use. H-151, a STING inhibitor, was purchased from Sigma-Aldrich (St. Louis, MO, USA) and also dissolved in DMSO and stored at -80 °C. Primary antibodies against various proteins, including glyceraldehyde-3-phosphate dehydrogenase (GAPDH), poly ADP ribose polymerase (PARP), Phospho-STING (Ser366), Phospho-TBK1/NAK (Ser172) (D52C2), TBK1 (E8I3G), Phospho-IRF-3 (Ser386), and STING (#11904), were obtained from Cell Signaling Technology (CST; Danvers, MA, USA). Fluorescein-conjugated secondary antibodies (anti-rabbit and anti-mouse) were purchased from Odyssey (Lincoln, NE, USA). Annexin V/PI staining dye was acquired from BD Biosciences (San Jose, CA, USA). All other reagents and cell culture mediums were obtained from Thermo Fisher Scientific (Waltham, MA, USA), unless stated otherwise.

### 2.2 Cell lines and cell culture

The human NSCLC cell lines A549 and H358 cell lines were obtained from the American Type Culture Collection (ATCC, Virginia, USA). Both cell lines were cultured in RPMI-1640 medium manufactured by Gibco (Gibco, Grand Island, NY, USA). The medium was supplemented with 10% fetal bovine serum (FBS), 100 U/mL penicillin, and 100 μg/mL streptomycin (Sigma-Aldrich, St. Louis, MO, USA). All cell lines were maintained at 37 °C in a humidified atmosphere containing 5% CO_2_.

### 2.3 Cytotoxicity assay

Cell viability was assessed using the cell counting kit-8 (CCK8; Dojindo, Kumamoto, Japan) method, as described previously (25). A549 and H358 cells were seeded in 96-well plates at 3000 cells/well and treated with different concentrations of PTE (1.25, 2.5, 5, 10, and 20 µM) for 24, 48, and 72 hours. After incubation, 10 µL of CCK-8 reagent was added to each well and incubated for 4 hours. Absorbance was measured at 450 nm using a microplate reader (BioTek, Winooski, VT, USA). All experiments were performed with at least three independent replicates (n=3).

### 2.4 Apoptosis analysis by flow cytometry

Apoptosis was evaluated using an Annexin V-FITC/propidium iodide (PI) Apoptosis Detection Kit (BD Biosciences, San Jose, CA, USA) (26). Cells were treated with PTE, N-acetylcysteine (NAC), PTE, H-151 or corresponding vehicles for 24 hours, harvested, and stained according to the manufacturer’s protocol. Flow cytometry was performed using a BD FACSAria™ III cell sorter (BD Biosciences), and data were analyzed with FlowJo software.

### 2.5 Cell cycle analysis by flow cytometry

Cells were harvested, washed with PBS, and fixed in 70% ice-cold ethanol at -20 °C for at least 4 hours. Fixed cells were pelleted, treated with RNase A (100 µg/mL) at 37 °C for 30 minutes to digest RNA, and then stained with propidium iodide (PI, 50 µg/mL) in the dark at room temperature for 15 minutes before analysis (27, 28). Cell cycle distribution was analyzed using a BD FACSAria III flow cytometer. was analyzed using a BD FACSAria III flow cytometer and data were analyzed with Flowjo software.

### 2.6 Transwell assay

Cell invasion was assessed using 8 µm pore-size Transwell chambers (Corning, Corning, NY, USA). Briefly, 1 x 10^4^ cells in 200 µL serum-free medium were seeded into the upper chamber, with or without PTE (0-30 µM) or cisplatin (6.67 µM). The lower chamber contained 600 µL complete medium. After 24 hours, non-invading cells were removed with a cotton swab. Invaded cells on the lower surface were fixed with paraformaldehyde for 20 minutes, stained with 0.1% crystal violet (Solarbio, Beijing, China) for 15 minutes at room temperature, and then washed. Membranes were imaged under an inverted microscope (IX73; Olympus, Tokyo, Japan) at 100x magnification and five random fields per membrane were counted.

### 2.7 Western blotting analysis

The protein extraction process followed an established protocol (29). Briefly, total protein was extracted from cells using RIPA lysis buffer (200 µL per well of a 6-well plate) supplemented with protease and phosphatase inhibitors. Protein concentration was determined using a BCA assay kit (Beyotime, China). Equal amounts of protein (30 µg) were separated by 8-12% SDS-PAGE and transferred to nitrocellulose membranes (Millipore, Billerica, MA, USA). After blocking with 5% non-fat milk in TBST for 1 hour at room temperature, membranes were incubated with primary antibodies (diluted 1:1000 in 5% BSA-TBST) overnight at 4 °C. The following primary antibodies were used: GAPDH, PARP, Phospho-STING (Ser366), Phospho-TBK1/NAK (Ser172), TBK1, Phospho-IRF-3 (Ser386), STING, and γ-H2AX (all from Cell Signaling Technology). After washing, membranes were incubated with IRDye-labeled secondary antibodies (LI-COR Biosciences, Lincoln, NE, USA; 1:15000 dilution) for 1 hour at room temperature. Protein bands were visualized using an Odyssey Infrared Imaging System (LI-COR).

### 2.8 Measurement of reactive oxygen species

Intracellular ROS levels were detected using the fluorescent probe DCFH-DA (Beyotime, Shanghai, China). After treatment with PTE for 24 hours, cells were incubated with 10 µM DCFH-DA for 30 minutes at 37 °C. Fluorescence intensity was measured by flow cytometry (30).

### 2.9 Colony formation assays

Cells were seeded into 6-well plates at 200 cells per well and treated with PTE (0-40 µM) or cisplatin (6.67 µM) for 24 hours. After 14 days, colonies were fixed and stained with 0.1% crystal violet. Visible colonies were counted manually (31).

### 2.10 Quantitative real-time PCR

Total RNA was extracted using TRIzol reagent (Invitrogen, Carlsbad, CA, USA) (24). cDNA was synthesized using a PrimeScript RT reagent kit (Takara, Dalian, China). qPCR was performed with SYBR Green Premix (Takara) on a QuantStudio 5 Real-Time PCR System (Applied Biosystems, Foster City, CA, USA). The primers used for the qPCR were consistent with those reported in prior literature to ensure the reliability and comparability of the results (32).

### 2.11 Transient transfection assay

Cells were seeded in 6-well plates and grown to 60-70% confluence. Transfected was performed using Lipofectamine 3000 reagent (Invitrogen) according to the manufacturer’s instructions. For each well, 2 ug of control shRNA or STING-specific shRNA using lentiviral vectors (Santa Cruz Biotechnology, Dallas, TX, USA) was mixed with the transfection reagent and added to the cells. The medium was replaced with fresh complete medium 6 hours post-transfection. Knockdown efficiency was verified by Western blot and qPCR after 72 hours.

### 2.12 Xenograft study

All animal procedures were approved by the Animal Ethics Committee of Zhuhai Hospital of Integrated Traditional Chinese & Western Medicine (Approval No. EC-C-008-A04-V1.0) and were conducted in accordance with the National Institutes of Health Guide for the Care and Use of Laboratory Animals (33). This study is reported in accordance with the ARRIVE guidelines. Eight-week-old male BALB/c nude mice were subcutaneously inoculated with 5×10^5^ H358 cells suspended in serum-free media into the right flank (31). When tumor volumes reached 50 to 100 mm^3^, mice were randomly divided into experimental groups, with five mice in each group. For 21 days, mice received daily intraperitoneal injections of either a carrier solution consisting of 30% polyethylene glycol 400 (PEG400) and 5% Tween 80 in PBS or PTE at a concentration of 10 mg/kg, also dissolved in the same carrier solution. Throughout the treatment period, the overall health of the animals was monitored, including weight fluctuations and any signs of toxicity, such as >20% body weight loss, lethargy, hunched posture, ruffled fur, or reduced mobility, which were not observed in our study.

Tumor growth was measured every three days using calipers, and tumor volume was calculated using the formula: volume = length (mm) × width (mm)² × π/6 (34). At the end of the treatment period, mice were euthanized via exposure to carbon dioxide (CO_2_) in a sealed chamber at a flow rate of 20% chamber volume per minute (until respiratory arrest was confirmed). The tumors were then surgically excised from the mice for further analysis. These procedures ensured effective monitoring and evaluation of tumor growth and the impact of PTE treatment on the xenografts, all in accordance with animal welfare guidelines.

### 2.13 Detection of anti-tumor immune response to PTE

The effect of PTE on the anti-tumor immune response was evaluated in an 8-week-old male C57BL/6 mouse model transplanted with LLC1 cells, which establishes a fully immunized lung cancer scenario (35). Mice received subcutaneous injections of LLC1 cells, and upon tumors reaching sizes of 50 to 100 mm³, they were randomly assigned to either the solvent (control) group or the PTE treatment group for a duration of 21 days. Mice were euthanized when the tumor diameter reached 15 mm (predefined humane endpoint) via exposure to carbon dioxide (CO_2_) in a sealed chamber at a flow rate of 20% chamber volume per minute (until respiratory arrest was confirmed). After euthanasia, both tumor weight and volume were measured.

Single-cell suspensions were prepared from the tumors. The following anti-mouse antibodies were used: PerCP anti-mouse CD45 Antibody (clone 30-F11), APC anti-mouse CD3 antibody (clone 17A2), PE-cy7 anti-mouse CD8a antibody (clone 53-6.7), FITC anti-mouse CD4 Antibody (clone RM4-5), PE anti-mouse FOXP3 antibody (clone MF-14), Gr-1 (clone RB6-8C5), APC/Cyanine7 anti-mouse/human CD11b Antibody (clone M1/70), PE/Cyanine7 anti-mouse F4/80 Antibody (clone BM8) from BioLegend or eBioscience. For cytokine detection, cells were stimulated with Cell Stimulation Cocktail (plus protein transport inhibitors, eBioscience) for 4 hours, followed by staining for PE/Dazzle™ 594 anti-mouse TNF-α antibody (clone MP6-XT22), APC/Cy7 anti-mouse IFN-γ antibody (clone XMG1.2), and Granzyme B (clone NGZB). The levels of TNF-α, GrzmB, and IFN-γ in tumor-infiltrating CD8^+^ T cells were quantified by measuring the Mean Fluorescence Intensity (MFI) and the percentage of positive cells using flow cytometry (BD FACSAria™ III) and analyzed with FlowJo software. The study specifically focused on the impact of PTE on tumor-infiltrating CD8^+^ T cells, assessing the levels of TNF-α^+^, granzyme B^+^, and IFN-γ^+^ within these cells. Tumor and spleen tissues were homogenized, single cells were isolated, and flow cytometry analysis was performed following treatment with anti-CD16/32 antibodies and staining with specific antibodies per the manufacturer’s instructions. The resulting cell populations were analyzed via flow cytometry.

Additionally, we evaluated the composition of immune cells in the TME post-PTE treatment, including Tregs, DCs, macrophages, and infiltrating CD8^+^ T cells. The levels of TNF-α, granzyme B (GrzmB), and IFN-γ in tumor-infiltrating CD8^+^ T cells were quantified to further understand the immune responses induced by PTE.

### 2.14 Statistical analysis

The data presented in the study are expressed as the mean ± standard deviation (SD) from three independent experiments. Statistical differences between groups were evaluated using one-way analysis of variance (ANOVA), followed by Bonferroni’s test for pairwise comparisons. Results were deemed statistically significant at the following thresholds: *P < 0.05, **P < 0.01, ***P < 0.001, and ****P < 0.0001, indicating the level of significance for the observed differences among the experimental groups.

## 3 Results

### 3.1 PTE significantly inhibited the viability of NSCLC cells

**Figure 1:**
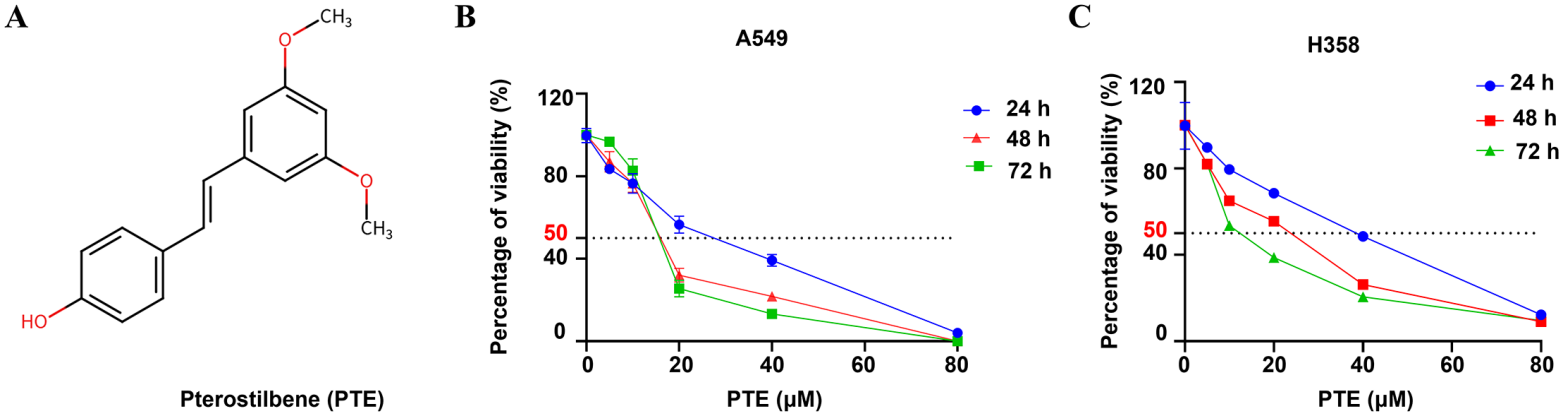
PTE inhibited growth in NSCLC cells. **(A)** The structure of the compound pterostilbene (PTE) is illustrated. **(B, C)** To evaluate the effects of PTE on the viability of NSCLC cell lines A549 and H358, cells were treated with varying concentrations of PTE (0, 1.25, 2.5, 5, 10, 20, 40 µM) for 24, 48, and 72 hours. Cell viability was then assessed using CCK8 assays, which measure metabolic activity as an indicator of cell viability. The results indicated a concentration- and time-dependent response to PTE treatment in both cell lines. Data are presented as mean ± SD from three independent experiments (n=3). Statistical significance is denoted as *P < 0.05, **P < 0.01, and ***P < 0.001.

The chemical structure of pterostilbene (PTE) is shown in **Figure 1A**. We evaluated its effects on cell viability using CCK-8 assays in A549 and H358 NSCLC cells treated with various concentrations of PTE (0-40 µM) for 24, 48, and 72 hours. PTE significantly inhibited cell viability in both cell lines in a concentration- and time-dependent manner (**Figures 1B, C**).

The calculated half-maximal inhibitory concentration (IC_50_) values indicated differing sensitivities to PTE between the two cell lines. Specifically, the IC_50_ values were 31.79 µM for A549 and 68.3 µM for H358 at the 24-hour mark, 7.46 µM and 29.3 µM at 48 hours, and 4.66 µM and 6.67 µM at 72 hours, respectively. Based on these findings and to guide future experiments, we selected concentrations of 10 µM, 20 µM, 30 µM, and 40 µM for a 24-hours treatment period as the experimental conditions for further investigations.

### 3.2 PTE effectively suppressed both the proliferation and invasion of NSCLC cells

**Figure 2:**
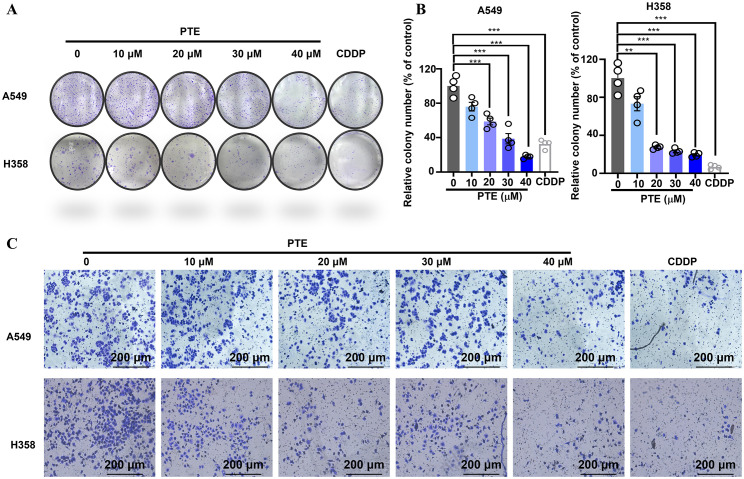
PTE suppressed both the proliferation and invasion of NSCLC cells. **(A, B)** The results of the colony formation assay demonstrated the impact of PTE treatment (0, 10, 20, 30, 40 μM) on the number of colonies formed by NSCLC cell lines A549 and H358. Cisplatin (CDDP, 6.67 μM) was used as a positive control to compare the proliferative effects of PTE. **(C)** Cells were treated with PTE (0, 10, 20, 30, 40 μM) or cisplatin (CDDP, 6.67 μM) and then assessed for their ability to invade through a Matrigel matrix using Transwell assay Additionally, the assessment of cell invasion revealed that PTE treatment significantly impacted the invasive capabilities of the cancer cells in a concentration-dependent manner. Data are presented as mean ± SD from three independent experiments (n=3).Statistical significance is indicated as *P < 0.05, **P < 0.01, ***P < 0.001, and ****P < 0.0001.

The colony formation assay is a crucial method for assessing the long-term growth potential of cancer cells (36). Colony formation assays revealed that PTE treatment for 14 days significantly reduced the number of colonies in A549 and H358 cells in a concentration-dependent manner, with efficacy comparable to cisplatin (6.67 µM; **Figures 2A, B**).

Transwell invasion assays further demonstrated that PTE suppressed cell invasion in a dose-dependent fashion (**Figure 2C**). These findings indicated that PTE restrained both proliferative capacity and invasive behavior in NSCLC cells.

### 3.3 PTE induced cell apoptosis in NSCLC cells

**Figure 3:**
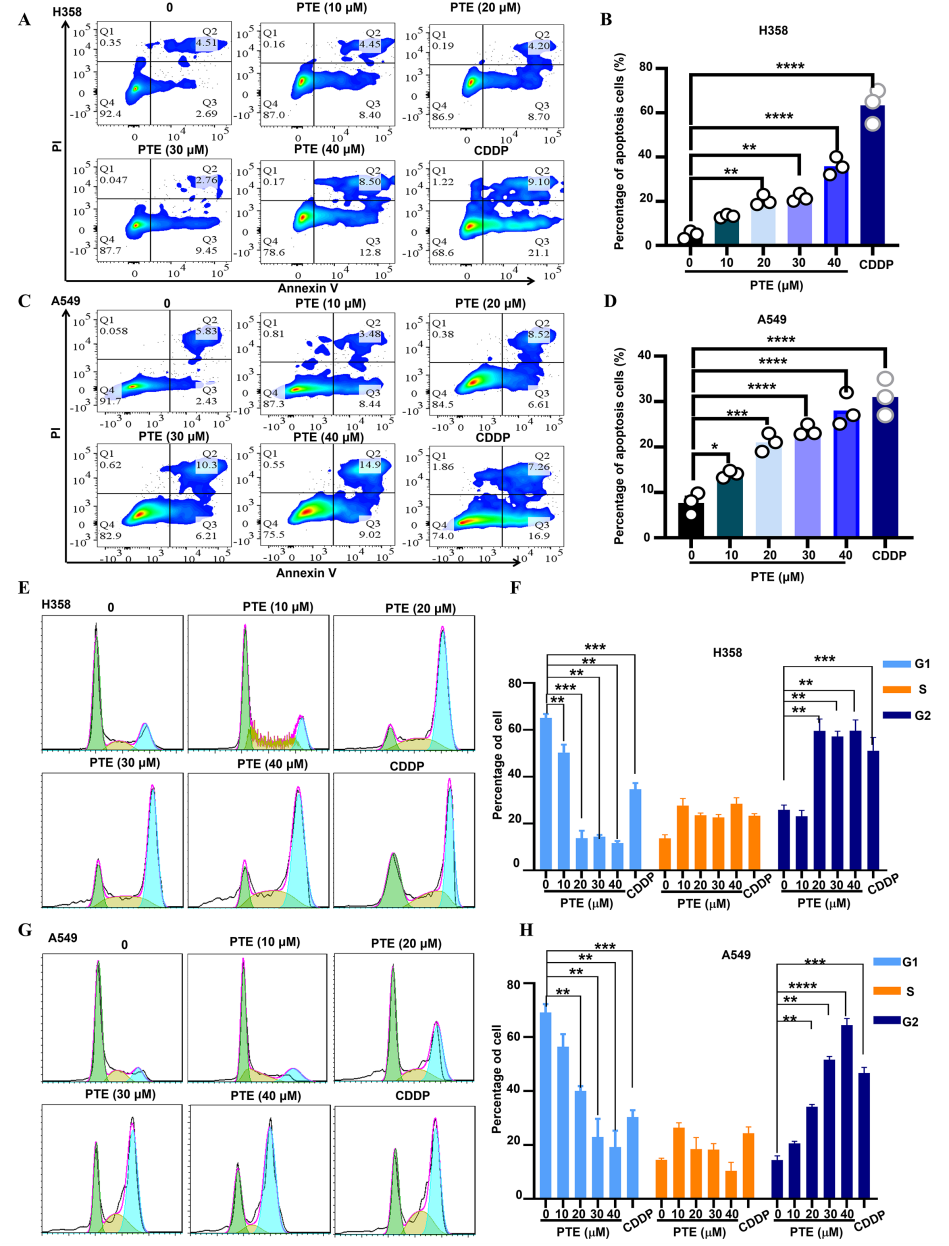
PTE exhibited a significant increase apoptosis in NSCLC cells. **(A-D)** A549 and H358 cells were treated with PTE at concentrations of 10, 20, 30 and 40 μM for 24 hours. The analysis of cell apoptosis in these NSCLC cells was conducted using BD Annexin V/PI staining followed by flow cytometry, demonstrating the apoptotic effects of PTE treatment. **(E-H)** The impact of PTE on cell cycle progression was also assessed in A549 and H358 cells after 24-hours treatments. Cells were stained with PI and analyzed with flow cytometry to determine cell cycle arrest. Data are presented as the percentage of cells in each phase of the cell cycle (G1, S, and G2/M phases). Statistical significance is indicated by *P < 0.05, **P < 0.01, ***P < 0.001 and **** *P* < 0.0001.

Using Annexin V/PI staining and flow cytometry, we observed that PTE treatment induced apoptosis in A549 and H358 cells in a concentration-dependent manner (**Figures 3A–D**). Additionally, cell cycle analysis showed that PTE treatment resulted in G2/M phase arrest (**Figures 3E–H**). These data suggested that PTE promoted apoptotic cell death and disrupted cell cycle progression in NSCLC cells.

### 3.4 PTE induced ROS production in NSCLC cells

**Figure 4:**
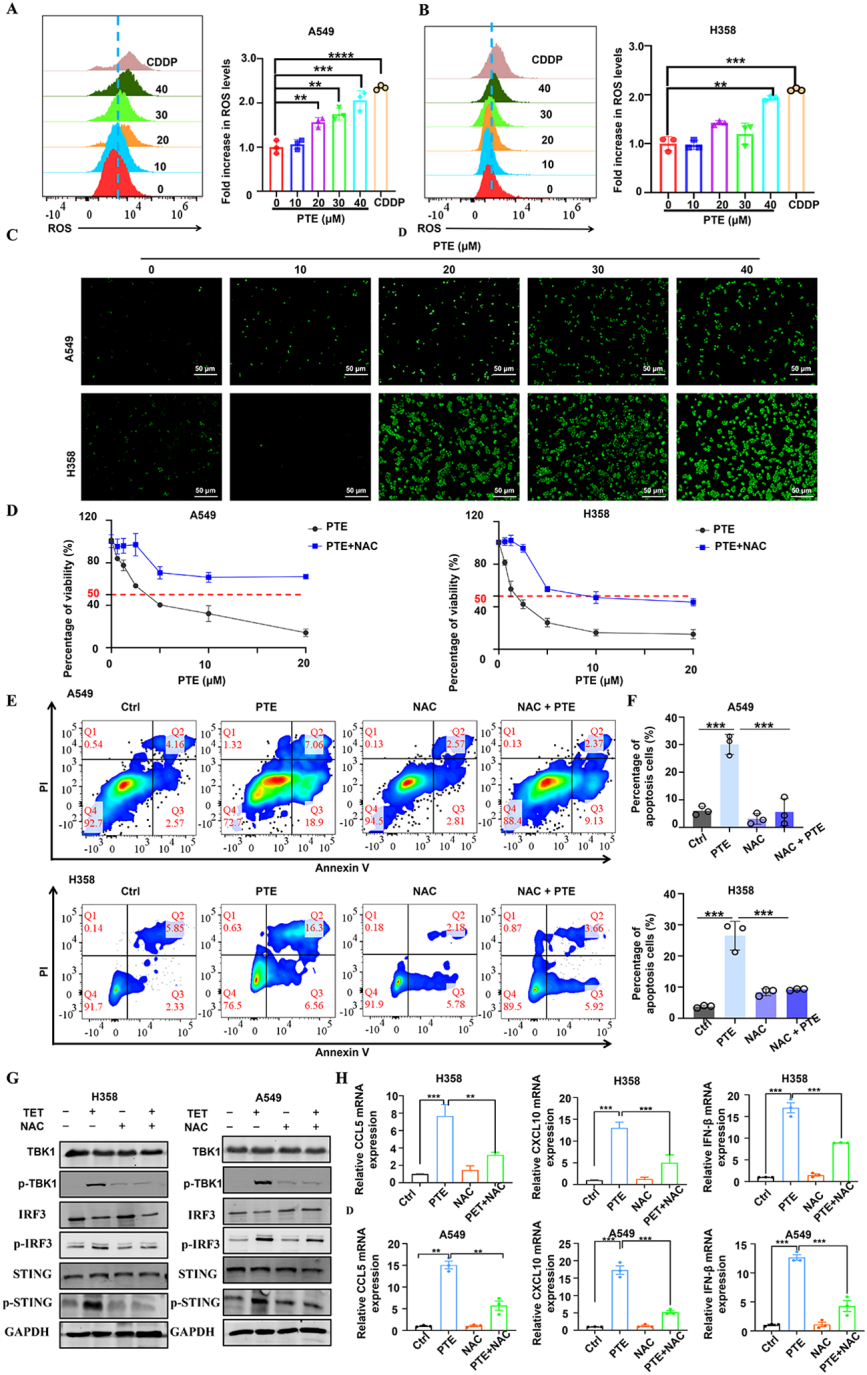
PTE induced mitochondrial dysfunction and induced ROS production in NSCLC cells. **(A, B)** A549 and H358 cells were treated with PTE for 24 hours, and ROS levels were measured using DCFDA staining followed by flow cytometry analysis, indicating a significant increase in ROS levels. **(C)** The expression of reactive oxygen species (ROS) in A549 and H358 cell lines was further evaluated using immunofluorescence techniques after 24 hours of PTE treatment, confirming elevated ROS levels. **(D)** A549 and H358 cells were pre-incubated with 5 mM NAC for 1 hour prior to treatment with PTE for 24 hours, and cell proliferation was assessed using the CCK8 assay, demonstrating that NAC pretreatment reversed the inhibitory effects of PTE. **(E, F)** Additionally, A549 and H358 cells were pre-incubated with 5 mM NAC for 1 hour before PTE treatment for 24 hours, and cell apoptosis was evaluated using flow cytometry, further elucidating the role of ROS in PTE-induced apoptosis. **(G)** A549 and H358 cells were pre-incubated with 5 mM NAC for 1 hour before PTE treatment for 24 hours. Western blot analysis was conducted to assess the protein expression levels of p-STING, STING, p-TBK1, TBK1, p-IRF3, IRF3, and GAPDH. **(H)** A549 and H358 cells were pre-incubated with 5 mM NAC for 1 hour before PTE treatment for 24 hours, mRNA expression levels of C-C motif chemokine ligand 5 (CCL5), C-X-C motif chemokine ligand 10 (CXCL10), C-X-C motif chemokine ligand 9 (CXCL9), and interferon beta (IFN-β) was examined with RT-PCR. Data were presented as the mean ± S.D. from three independent experiments. *P < 0.05, **P < 0.01, ***P < 0.001 and ****P < 0.0001.

Reactive oxygen species (ROS) serve as key mediators of apoptosis (37). Prior research has indicated that PTE induces apoptosis in glioma cells via ROS production (38). In this study, we evaluated ROS levels using the fluorescent probe DCFDA. Flow cytometry analysis revealed a significant increase in ROS levels in both A549 and H358 NSCLC cells following PTE treatment (**Figures 4A, B**). Furthermore, immunofluorescence data supported these findings, showing elevated ROS levels as a result of PTE treatment (**Figure 4C**).

To investigate the relationship between ROS and PTE-induced growth inhibition, we conducted experiments with the ROS scavenger NAC. The CCK8 assay demonstrated that pretreatment with NAC effectively reversed the inhibitory effects of PTE on cell proliferation in both A549 and H358 cells (**Figure 4D**). Additionally, NAC pretreatment significantly reduced PTE-induced apoptosis (**Figures 4E, F**). Collectively, these findings indicated that ROS played a pivotal role in PTE-induced cell death and the inhibition of cell proliferation in NSCLC cells, highlighting the potential of targeting ROS pathways in cancer therapy.

The STING pathway plays a critical role in modulating the TME by regulating both antitumor immunity and cellular proliferation (25, 39). To clarify the relationship between ROS and STING activation, we conducted additional experiments in which cells were pretreated with NAC followed by PTE treatment. Western blot analysis showed that NAC pretreatment markedly suppressed PTE-induced phosphorylation of STING, TBK1, and IRF3 (**Figure 4G**). Additionally, NAC abolished PTE-induced upregulation of CXCL10 and CCL5 mRNA, as measured by qPCR (**Figure 4H**). These results confirmed that ROS generation preceded and was required for STING pathway activation and subsequent chemokine production.

### 3.5 PTE significant activated STING pathway in NSCLC cells

**Figure 5:**
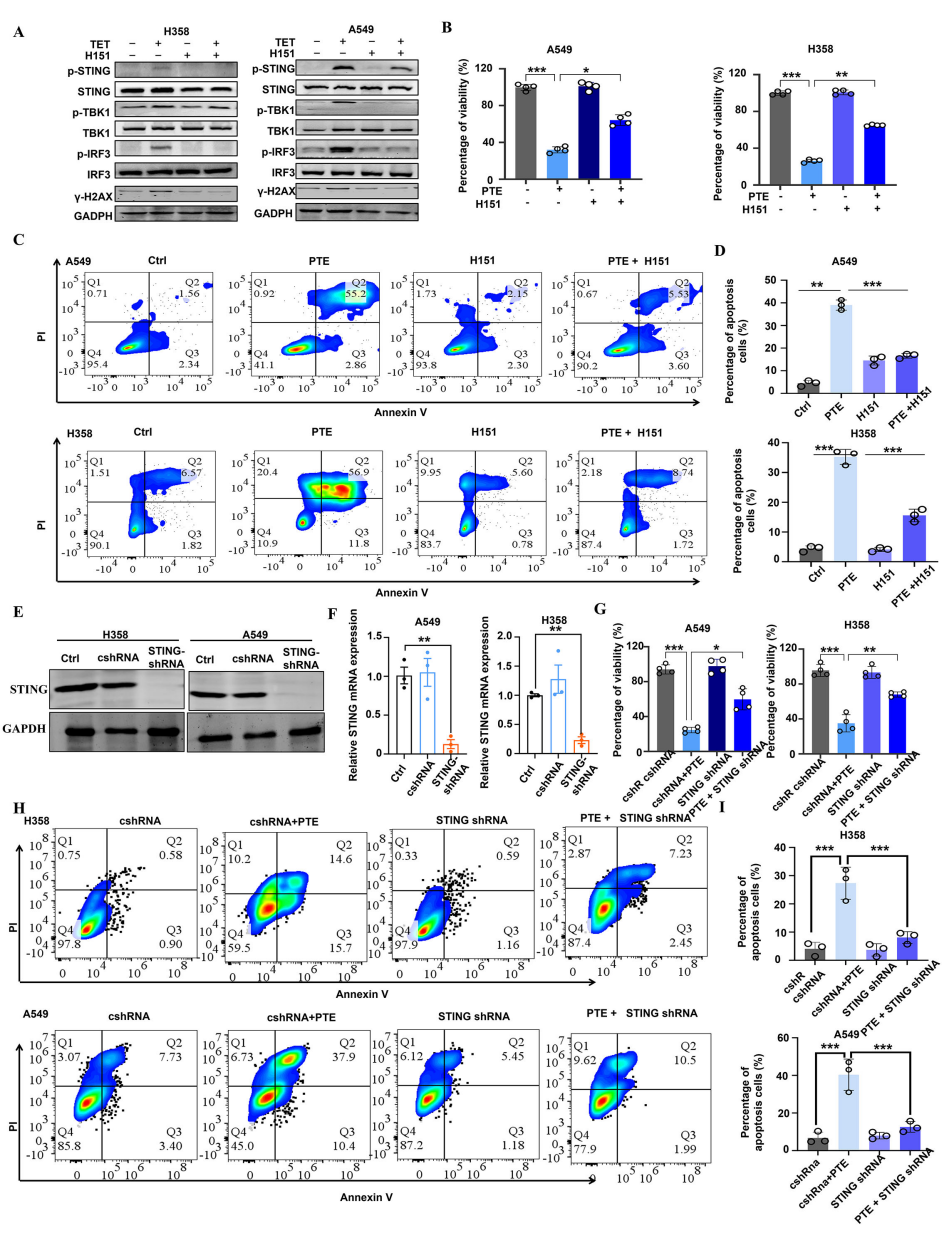
PTE significantly activated the STING pathway in NSCLC. **(A)** A549 and H358 cells were pre-incubated with H-151 for 1 hour prior to treatment with PTE for 24 hours. Western blot analysis was conducted to assess the protein expression levels of p-STING, STING, p-TBK1, TBK1, p-IRF3, IRF3, and GAPDH. **(B)** Following the same pre-incubation with H-151 for 1 hour, the cell proliferation of A549 and H358 cell lines was evaluated using the CCK-8 assay after 24 hours of PTE treatment. **(C, D)** Cell apoptosis in A549 and H358 cells was analyzed using flow cytometry following the preincubation with H-151 and subsequent PTE treatment for 24 hours. **(E)** The Western blot assay results show that STING was successfully knocked down in H358 and A549 cells. **(F)** RT-PCR assay showed that STING mRNA expression was decreased in H358 and A549 cells after treated with STING shRNA. **(G)** The cell viability of H358 and A549 cells subjected to the knockdown of STING with the indicated doses of PTE treatment for 48 h was assessed with the MTT method. **(H, I)** Cell apoptosis in A549 and H358 cells subjected to the knockdown of STING with the indicated doses of PTE treatment for 48 hours was assessed with using flow cytometry. Data were presented as the mean ± S.D. from three independent experiments. *P < 0.05, **P < 0.01, ***P < 0.001 and ****P < 0.0001.

To determine whether STING signaling contributes to the antitumor effects of PTE, we examined pathway activation in A549 and H358 cells after PTE treatment. As shown in **Figure 5A**, PTE treatment markedly increased phosphorylation of STING, TBK1, and IRF3, indicating activation of the pathway.

STING activation is commonly triggered by cytoplasmic double-stranded DNA (dsDNA), often resulting from genomic stress or DNA damage (39, 40). We therefore evaluated DNA damage responses by measuring γ-H2AX expression. Elevated levels of γ-H2AX were detected in both cell lines following PTE treatment, confirming induction of DNA damage (**Figure 5A**). To further investigate the functional role of STING in PTE-induced effects, we used the selective STING inhibitor H-151 (41). H-151 pretreatment significantly suppressed PTE-induced phosphorylation of TBK1 and IRF3 (**Figure 5A**), and partially restored cell proliferation (**Figure 5B**) and viability (**Figures 5C, D**), supporting the functional involvement of STING in PTE-mediated anticancer activity.

To complement the pharmacological inhibition approach and rule out off-target effects of H151, we performed shRNA-mediated knockdown of STING in A549 and H358 cells. Western blot analysis confirmed successful reduction of STING protein expression in both cell lines (**Figure 5E**), and qPCR verified the downregulation of STING mRNA (**Figure 5F**). Consistent with the H-151 results, STING knockdown significantly reversed PTE-induced inhibition of cell viability (**Figure 5G**) and reduced PTE-induced apoptosis (**Figures 5H, I**). These convergent findings from both pharmacological and genetic approaches strongly confirmed that STING activation was essential for the antitumor effects of PTE. Collectively, these results suggest that PTE effectively activated the STING pathway in NSCLC cells.

### 3.6 PTE inhibited H358 tumor *xenograft* growth

**Figure 6:**
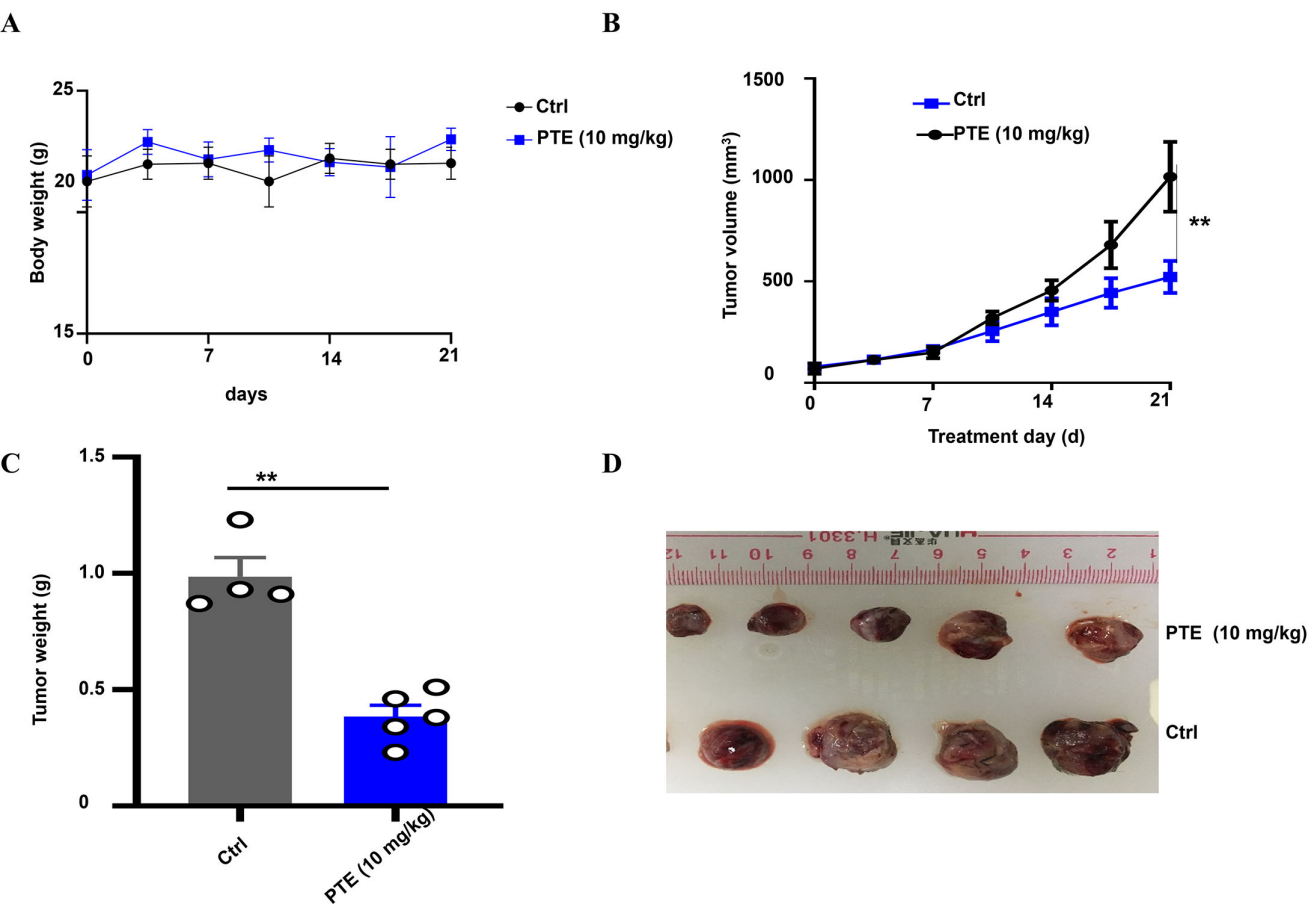
PTE inhibited NSCLC tumor *xenografts*. **(A)** Body weight of mice bearing NSCLC xenografts was monitored throughout the treatment period to assess overall health and effects of different treatments. Body weights are presented in grams (g). **(B)** Graph showing tumor volume over time, demonstrating significant inhibition of tumor growth in the PTE treatment group compared to controls from day 14 onward. **(C)** Representative images of tumors harvested from both groups highlight the smaller size of tumors in the PTE treatment group. **(D)** Data presenting the weight of tumors from both groups further confirmed the reduced tumor burden in the PTE-treated mice. The data presented represent the mean values along with standard deviations derived from three separate experiments. ^*^ *P* < 0.05, ^**^ *P* < 0.01, ^***^ *P* < 0.001.

To evaluate the anticancer efficacy of PTE *in vivo*, we established a *xenograft* model by subcutaneously inoculating H358 cells into male BALB/c nude mice (42, 43). The mice were randomly assigned to two groups: one treatment group that received PTE (10 mg/kg) and a control group administered with the vehicle solution (5% DMSO, 10% Tween 80, 30% PEG400, and 55% normal saline). Both solutions were delivered via daily intraperitoneal injection. Throughout the experimental period, no significant differences in body weight were observed between the PTE -treated and control groups (**Figure 6A**), suggesting that PTE did not induce systemic toxicity at the administered dose.

Notably, PTE treatment significantly suppressed tumor growth compared to the vehicle control. A statistically significant reduction in tumor volume became apparent from day 14 post-treatment onward (**Figure 6B**). At the endpoint, tumors resected from the PTE -treated group were both visibly smaller (**Figure 6C**) and had significantly lower weights than those from the control group (**Figure 6D**). These consistent results across volume and weight measurements robustly demonstrate the inhibitory effect of PTE on NSCLC tumor progression *in vivo*. In summary, these findings indicated that PTE effectively restrained tumor growth in a *xenograft* model without eliciting overt adverse effects, underscoring its potential as a promising therapeutic candidate for NSCLC treatment.

### 3.7 PTE inhibited tumor growth by increasing CD8^+^ T cells in mouse NSCLC model

**Figure 7:**
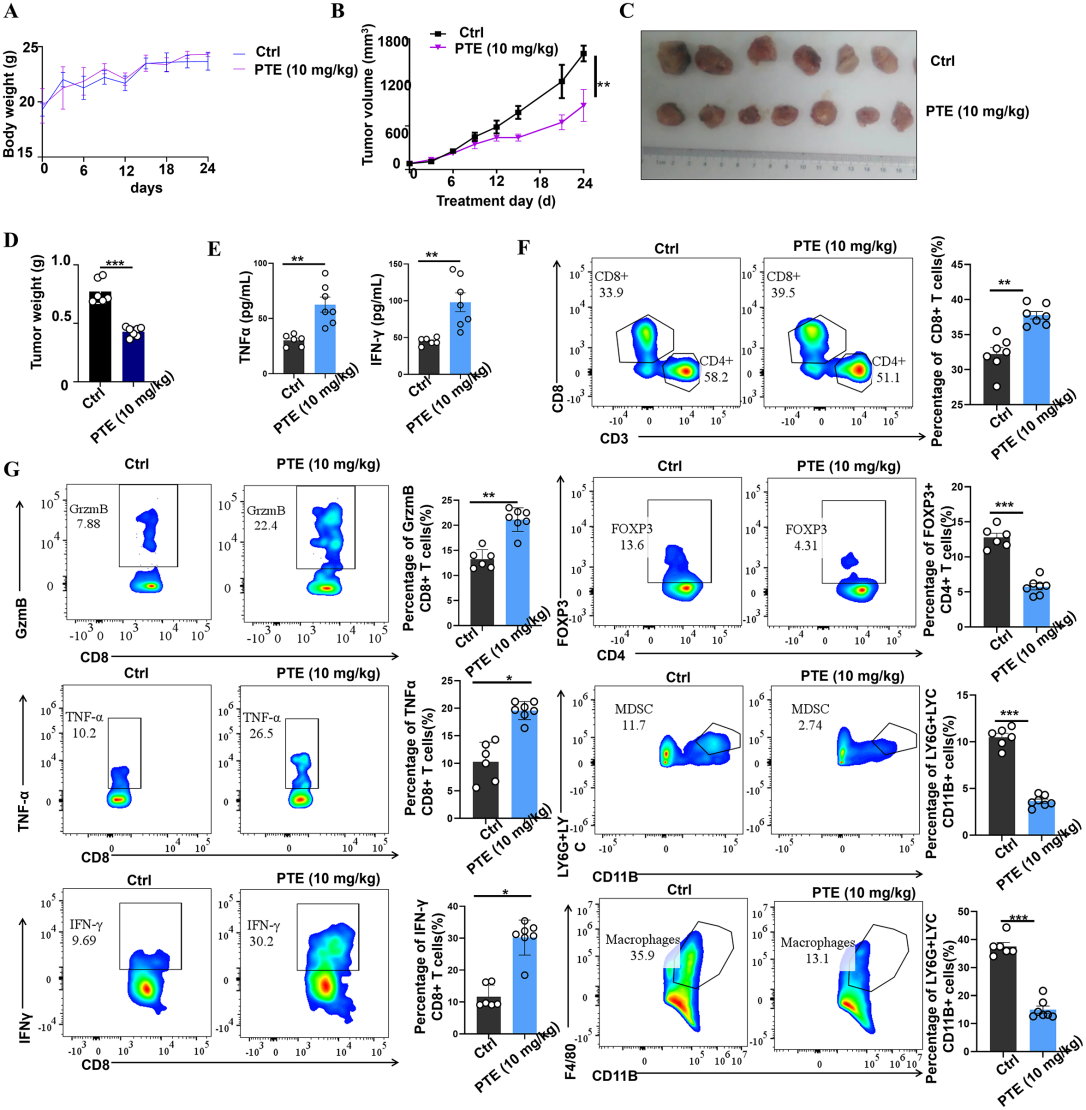
PTE treatment was found to inhibit tumor growth by enhancing the presence of CD8+ T cells *in vivo*. **(A)** Body weight change curve of NSCLC mice treated with either vehicle or PTE, showing no significant weight loss in the PTE-treated group. **(B)** Tumor volume in the NSCLC mouse model was evaluated from the beginning of the treatment period, illustrating tumor suppression in the PTE-treated group compared to the control. **(C)** Photographs of tumor samples from each group, highlighting the reduced size of tumors in the PTE treatment group. **(D)** Measurement of tumor weights in NSCLC mice on day 24 post-treatment, confirming significant reduction in tumor weight in the PTE group relative to the control group. **(E)** ELISA results displaying quantified levels of TNF-α and IFN-γ in plasma, indicating elevated cytokine levels following PTE treatment. **(F)** Flow cytometry analysis demonstrating the distribution of CD8+ T cells in tumor tissues. **(G)** Flow cytometry analysis demonstrating the distribution of CD8+ T cells in tumor tissues and assessing the expression of TNF-α, granzyme B (GzmB), and IFN-γ among CD8+ T cells, along with the evaluation of Tregs, MDSCs, and tumor-infiltrating macrophages in control versus PTE treatment groups. Flow cytometric data was compared among the groups, with statistical significance denoted by P values (*P < 0.05, **P < 0.01, ***P < 0.001, and ****P < 0.0001).

Activation of the STING pathway in tumor cells can initiate potent innate and adaptive immune response against tumors (44). To investigate whether PTE exerts its anticancer effects through immunomodulation, we utilized an immunocompetent murine model of NSCLC. Treatment with PTE did not cause significant weight loss in NSCLC mice, as shown by the body weight change curve (**Figure 7A**). From day 15 of treatment onward, we observed a notable trend of tumor suppression in the PTE treatment group (**Figure 7B**), accompanied by significant reductions in tumor volume (**Figures 7B, C**) and overall tumor weight (**Figure 7D**).

To elucidate the immune mechanisms involved, we first measured systemic levels of key antitumor cytokines. At the endpoint of the experiment (day 24), ELISA analysis of mouse plasma revealed that PTE treatment significantly elevated concentrations of IFN-γ and TNF-α (**Figure 7E**), suggesting activation of a Th1-type immune response. To gain deeper insights into the enhanced therapeutic response, we employed flow cytometry to evaluate the anti-tumor immune activity within the TME, and **Supplementary Figures S1**, **2** showed the flow cytometric analysis gating strategy. Our analysis revealed an increase in both the activity and abundance of CD8^+^ T cells in the PTE-treated group compared to the control group receiving carrier solution (**Figure 7F**). Notably, CD8^+^ T cells in the tumors of PTE-treated mice exhibited a more robust activation profile, producing significantly higher levels of TNF-α, GrzmB, and IFN-γ compared to the control group (**Figure 7G**).

Moreover, we observed a reduction in the populations of MDSCs, Tregs, and TAMs in the tumors of PTE-treated mice. This finding suggests that PTE not only enhances the activation of anti-tumor CD8^+^ T cells but also alleviates the suppressive immune environment in the TME. Overall, these results indicate that PTE treatment enhances the immune response and significantly improves the functionality of CD8^+^ T cells, thus promoting tumor control and survival in mouse models of NSCLC. Furthermore, PTE’s ability to reduce the expression of Tregs, MDSCs, and tumor-infiltrating macrophages underscores its potential as an effective therapeutic strategy for NSCLC.

## 4 Discussion

In this paper, we demonstrate that PTE effectively inhibits NSCLC progression through a dual mechanism involving direct ROS-mediated cytotoxicity and STING-dependent immune activation. Our key findings are: (1) PTE induces ROS-dependent DNA damage, cell cycle arrest, and apoptosis; (2) PTE activates the STING/TBK1/IRF3 pathway in a ROS-dependent manner; (3) Genetic and pharmacological inhibition of STING attenuates PTE’s effects, establishing its necessity; (4) PTE inhibits tumor growth in *xenograft* models; and (5) In an immunocompetent model, PTE enhances CD8^+^ T cell infiltration and function while reducing immunosuppressive populations.

Lung cancer remains the most prevalent malignancy and leading cause of cancer-related deaths worldwide (45, 46). While treatment options for advanced NSCLC have expanded to include chemotherapy, targeted therapy, immunotherapy, radiation therapy, and combination therapies (47), significant challenges persist including severe side effects and limited efficacy, particularly in patients with KRAS mutations who frequently develop drug resistance (48, 49). There is consequently a pressing need for novel therapeutic agents (46, 50) and natural compounds from traditional medicine are gaining increasing attention for their potential in cancer therapy (51).

PTE, a naturally occurring methylated analog of resveratrol found in Pterocarpus marsupium and blueberries (21), exhibits a range of beneficial properties (52), including antioxidative, anti-inflammatory, and antitumor activities (53). While previous studies have reported anticancer effects across various malignancies including pancreatic (54), gastric (19), lung cancer (18). For example, research has shown that PTE suppresses cell growth and invasion in lung cancer by targeting COX (55). While the specific anticancer mechanisms of PTE in NSCLC remain largely unexplored, its diverse biological functions have garnered significant interest (56, 57). Interestingly, our findings revealed that PTE inhibited cell proliferation in NSCLC cells in a concentration-dependent manner and disrupted the cell cycle at the G2 phase in the A549 and H358 cell lines. NSCLC cells exhibit elevated mitochondrial bioenergetics associated with altered metabolic pathways. Our study demonstrated that PTE suppressed the mitochondrial respiratory chain in NSCLC cell lines.

Similar to many conventional chemotherapeutic agents that operate through ROS accumulation and mitochondrial disruption (58, 59), PTE appears to mediate its anticancer effects primarily through ROS generation. Moderate increases in ROS can suppress tumor progression by inducing DNA damage (60), whereas excessive ROS causes oxidative stress, leading to impaired proliferation and cell death (61). Thus, pharmacological modulation of ROS levels represents a promising strategy for selective anticancer therapy (62). Our results establish that ROS induction is a pivotal mechanism in PTE-induced NSCLC cell death, accompanied by elevated ROS levels.

Activation of the STING pathway has emerged as a promising immunotherapeutic strategy (44). We found that PTE activates STING signaling in NSCLC cells, likely through induction of DNA damage and increased cytosolic double-stranded DNA (dsDNA), both known triggers of this pathway (63). This mechanism aligns with reports that PARP inhibitors, CHK1 inhibitors (64), and conventional chemotherapeutics like cisplatin and gemcitabine can activate STING through cytosolic dsDNA accumulation in various cancers (39). Our data suggest that PTE induces DNA damage and boosts cytosolic dsDNA, potentially initiating STING activation in NSCLC.

The TME plays a crucial role in NSCLC treatment response. Although CD8^+^ T cells are crucial for antitumor immunity, the NSCLC TME exhibits immunosuppressive characteristics that hinder T cell function (13). Thus, strategies to enhance antitumor immunity frequently focus on countering immunosuppressive elements (65), such as by targeting Tregs or MDSCs (66). Our results suggest that PTE modulates the TME favorably by promoting CD8^+^ T cell infiltration and reducing immunosuppressive cell populations (67–69).

The development of combination therapies represents a pivotal direction in improving cancer immunotherapy outcomes (70–72). Based on our mechanistic findings-that PTE induces immunogenic cell death and activates the STING pathway-we hypothesize that PTE may synergize with immune checkpoint inhibitors targeting the PD-1/PD-L1 axis. We speculate that PTE could prime the TME by recruiting T cells via chemokine induction, potentially enabling more effective reversal of T cell exhaustion by anti-PD-1/PD-L1 agents. This combination merits further exploration as a potential strategy to overcome resistance in immunologically cold tumors with minimal T cell infiltration.

The critical role of ROS in PTE-induced apoptosis was confirmed by the ability of NAC to attenuate this effect. *In vivo*, PTE administration reduced tumor weight and volume in H358 xenograft models and significantly suppressed tumor growth in immunocompetent NSCLC models, correlating with increased intratumoral CD8^+^ T cell accumulation. Together, these results illuminate a novel mechanism through PTE restrains NSCLC growth via ROS induction and STING pathway activation.

### 4.1 Pharmacokinetic properties and translational potential of pterostilbene

Having established the mechanistic basis for PTE’s antitumor efficacy, we next considered its translational potential. Although the primary aim of this study was to elucidate the mechanistic basis of PTE’s antitumor effects, its translational potential justifies a discussion of pharmacokinetic properties. Previous reports indicate that PTE possesses superior pharmacokinetic profiles compared to resveratrol, attributable to its methoxyl groups, which enhance oral bioavailability and metabolic stability (73). Animal studies show that PTE reaches peak plasma concentrations within 15–30 minutes after oral administration and remains detectable for up to 8 hours, distributed in tissues including the lung, liver, and kidney (53). The dose used in our *in vivo* studies (10 mg/kg, intraperitoneal) yields plasma concentrations consistent with effective concentrations in our *in vitro* models, supporting the biological relevance of our findings. PTE has demonstrated a favorable safety profile in preclinical studies, with no significant toxicity observed at efficacious doses (74). In our hands, no significant weight loss or behavioral alterations were observed, suggesting limited systemic toxicity. We acknowledge that a detailed PK/PD study was beyond the scope of this initial mechanistic work. Future studies should focus on characterizing the pharmacokinetics, biodistribution, and maximum tolerated dose of PTE in immunocompetent NSCLC models to better bridge the gap between our findings and clinical application. Nano-formulation strategies, such as self-assembled micelles (75), could further improve tumor-specific delivery and minimize off-target effects.

### 4.2 Therapeutic window and combination potential with immunotherapies

Our proposed dosage of 10 mg/kg was based on prior studies demonstrating efficacy without overt toxicity. To contextualize PTE within the current NSCLC treatment landscape, we discuss its potential to overcome limitations of immune checkpoint inhibitors (ICIs). ICIs, such as anti-PD-1/PD-L1 agents, show limited efficacy in “immune-cold” tumors due to poor T-cell infiltration and immunosuppressive microenvironments (38). Our data suggest that PTE remodels the tumor microenvironment (TME) by activating STING-mediated signaling, leading to increased chemokine production (e.g., CXCL10, CCL5) and enhanced CD8^+^ T-cell recruitment and function. This mechanism aligns with recent strategies aiming to convert “cold” tumors into “hot” ones (48). We propose that PTE could synergize with ICIs by priming the TME—first, inducing immunogenic cell death and innate immune activation via ROS/STING, followed by ICI-mediated reversal of T-cell exhaustion. This sequence could potentially expand the therapeutic window and address ICI resistance. Future work will directly evaluate the efficacy of combining PTE with anti-PD-1 antibodies in immunocompetent NSCLC models. However, to meaningfully translate these preclinical insights, several key challenges and limitations of the current study must be addressed.

### 4.3 Limitations and future perspectives

Despite the promising findings, this study has several limitations that warrant acknowledgment. First, the *in vivo* models employed-including H358 *xenografts* in immunodeficient mice and the LLC1 model in immunocompetent mice-may not fully recapitulate the heterogeneity and immunosuppressive complexity of human NSCLC. The LLC1 model, in particular, is known to be relatively immunogenic (76), which might lead to an overestimation of PTE’s immunomodulatory efficacy. Future investigations should utilize genetically engineered mouse models (GEMMs) that better mimic key molecular subtypes of human NSCLC (e.g., KRAS-driven or KRAS/p53-mutant models (77)) to validate our findings in more clinically relevant settings. Second, while we established a crucial link between ROS and STING activation, the precise mechanistic steps, such as whether ROS primarily causes mitochondrial DNA release or sensitizes the STING pathway to existing cytosolic DNA, remain to be elucidated. Third, the causal relationship between STING activation, chemokine production, and antitumor immunity would be strengthened by functional validation experiments, such as *in vivo* CD8^+^ T cell depletion or neutralization of key chemokines like CXCL10 and CCL5.

To bridge the gap between our mechanistic insights and clinical application, we outline the following future research directions: (1) Detailed PK/PD and Toxicity Studies: Conduct comprehensive pharmacokinetic, biodistribution, and maximum tolerated dose studies of PTE in immunocompetent NSCLC models. It is crucial to evaluate advanced formulations (e.g., nano-micelles (75)) to enhance tumor targeting and minimize off-target effects. Ultimately, toxicity assessments in non-human primates would be invaluable for de-risking clinical translation. (2) Combination Therapy Trials: Systematically evaluate the synergistic potential of PTE with standard-of-care immune checkpoint inhibitors (e.g., anti-PD-1/PD-L1 antibodies) in a range of immunocompetent models. This should aim to verify our hypothesis that PTE can convert “immune-cold” tumors into “immune-hot” microenvironments (78), thereby overcoming a primary mechanism of ICI resistance. (3) Biomarker Development: Identify predictive biomarkers (e.g., tumor STING pathway activation status, baseline CD8^+^ T cell infiltration, or chemokine profiles) to help identify patient subgroups most likely to benefit from PTE-based therapies.

## 5 Conclusions

Our study demonstrates that PTE effectively inhibits NSCLC progression through dual mechanisms involving ROS-mediated cytotoxicity and STING-dependent immune activation. PTE significantly restrained tumor growth in both xenograft and immunocompetent models, correlating with enhanced CD8^+^ T cell infiltration and effector function. These findings nominate PTE as a promising therapeutic agent or adjunct for NSCLC treatment, particularly in combination with immune checkpoint inhibitors. Subsequent studies will directly interrogate the efficacy of combining PTE with anti-PD-1 antibodies in immunocompetent models to validate this therapeutic strategy.

## Data Availability

The original contributions presented in the study are included in the article/**Supplementary Material**. Further inquiries can be directed to the corresponding authors.

## Glossary

NSCLC: Non-small cell lung cancer
NAC: N-acetylcysteine
ICDs: Immune checkpoint inhibitors
PD-1: Programmed cell death 1
Tregs: regulatory T cells
TAMs: Tumor-associated macrophages
MDSCs: Myeloid-derived suppressor cells
ROS: Reactive oxygen species
GAPDH: glyceraldehyde-3-phosphate dehydrogenase
PARP: ADP ribose polymerase
PBS: Phosphate-buffered saline
PTE: Pterostilbene
PI3K: phosphatidylinositol 3-kinase
PI: propidium iodide
CDDP: Cisplatin
DCs: dendritic cells
PM: Pterocarpus marsupium
TKI: tyrosine kinase inhibitor
DMSO: dimethyl sulfoxide
ATCC: American Type Culture Collection
NC: nitrocellulose
qPCR: real-time fluorescent quantitative
PEG400: polyethylene glycol 400
dsDNA: double-stranded DNA
STING: Stimulator of Interferon Response
TBK1: TANK binding kinase 1
IRF3: Interferon regulatory Factor 3
CXCL10: C-X-C Motif Chemokine Ligand 10
CCL5: C-C Motif Chemokine Ligand 5
TKIs: Tyrosine kinase inhibitors

## References

[1] Bray F Laversanne M Sung H Ferlay J Siegel RL Soerjomataram I. Global cancer statistics 2022: GLOBOCAN estimates of incidence and mortality worldwide for 36 cancers in 185 countries. CA Cancer J Clin. (2024) 74:229–63. doi: 10.3322/caac.21834, PMID: 38572751

[2] Xia C Dong X Li H Cao M Sun D He S. Cancer statistics in China and United States, 2022: profiles, trends, and determinants. Chin Med J (Engl). (2022) 135:584–90. doi: 10.1097/CM9.0000000000002108, PMID: 35143424 PMC8920425

[3] Miller KD Nogueira L Devasia T Mariotto AB Yabroff KR Jemal A. Cancer treatment and survivorship statistics, 2022. CA Cancer J Clin. (2022) 72:409–36. doi: 10.3322/caac.21731, PMID: 35736631

[4] Anand U Dey A Chandel AKS Sanyal R Mishra A Pandey DK. Cancer chemotherapy and beyond: Current status, drug candidates, associated risks and progress in targeted therapeutics. Genes Dis. (2023) 10:1367–401. doi: 10.1016/j.gendis.2022.02.007, PMID: 37397557 PMC10310991

[5] Vasan N Baselga J Hyman DM. A view on drug resistance in cancer. Nature. (2019) 575:299–309. doi: 10.1038/s41586-019-1730-1, PMID: 31723286 PMC8008476

[6] Yuan M Huang LL Chen JH Wu J Xu Q. The emerging treatment landscape of targeted therapy in non-small-cell lung cancer. Signal Transduct Target Ther. (2019) 4:61. doi: 10.1038/s41392-019-0099-9, PMID: 31871778 PMC6914774

[7] Kraehenbuehl L Weng C-H Eghbali S Wolchok JD Merghoub T. Enhancing immunotherapy in cancer by targeting emerging immunomodulatory pathways. Nat Rev Clin Oncol. (2022) 19:37–50. doi: 10.1038/s41571-021-00552-7, PMID: 34580473

[8] Naimi A Mohammed RN Raji A Chupradit S Yumashev AV Suksatan W. Tumor immunotherapies by immune checkpoint inhibitors (ICIs); the pros and cons. Cell Commun Signal. (2022) 20:44. doi: 10.1186/s12964-022-00854-y, PMID: 35392976 PMC8991803

[9] Kong X Zhang J Chen S Wang X Xi Q Shen H. Immune checkpoint inhibitors: breakthroughs in cancer treatment. Cancer Biol Med. (2024) 21:451–72. doi: 10.20892/j.issn.2095-3941.2024.0055, PMID: 38801082 PMC11208906

[10] Horvath L Thienpont B Zhao L Wolf D Pircher A. Overcoming immunotherapy resistance in non-small cell lung cancer (NSCLC) - novel approaches and future outlook. Mol Cancer. (2020) 19:141. doi: 10.1186/s12943-020-01260-z, PMID: 32917214 PMC7488475

[11] Simula L Fumagalli M Vimeux L Rajnpreht I Icard P Birsen G. Mitochondrial metabolism sustains CD8+ T cell migration for an efficient infiltration into solid tumors. Nat Commun. (2024) 15:2203. doi: 10.1038/s41467-024-46377-7, PMID: 38467616 PMC10928223

[12] Lao L Zeng W Huang P Chen H Jia Z Wang P. CD8+ T cell-dependent remodeling of the tumor microenvironment overcomes chemoresistance. Cancer Immunol Res. (2023) 11:320–38. doi: 10.1158/2326-6066.CIR-22-0356, PMID: 36603133 PMC9975671

[13] Philip M Schietinger A. CD8+ T cell differentiation and dysfunction in cancer. Nat Rev Immunol. (2022) 22:209–23. doi: 10.1038/s41577-021-00574-3, PMID: 34253904 PMC9792152

[14] Li K Shi H Zhang B Ou X Ma Q Chen Y. Myeloid-derived suppressor cells as immunosuppressive regulators and therapeutic targets in cancer. Signal Transduction Targeted Ther. (2021) 6:362. doi: 10.1038/s41392-021-00670-9, PMID: 34620838 PMC8497485

[15] Rodriguez-Garcia A Lynn RC Poussin M Eiva MA Shaw LC O’Connor RS. CAR-T cell-mediated depletion of immunosuppressive tumor-associated macrophages promotes endogenous antitumor immunity and augments adoptive immunotherapy. Nat Commun. (2021) 12:877. doi: 10.1038/s41467-021-20893-2, PMID: 33563975 PMC7873057

[16] Christofides A Strauss L Yeo A Cao C Charest A Boussiotis VA. The complex role of tumor-infiltrating macrophages. Nat Immunol. (2022) 23:1148–56. doi: 10.1038/s41590-022-01267-2, PMID: 35879449 PMC10754321

[17] Tie Y Tang F Wei YQ Wei XW. Immunosuppressive cells in cancer: mechanisms and potential therapeutic targets. J Hematol Oncol. (2022) 15:61. doi: 10.1186/s13045-022-01282-8, PMID: 35585567 PMC9118588

[18] Bracht JWP Karachaliou N Berenguer J Pedraz-Valdunciel C Filipska M Codony-Servat C. Osimertinib and pterostilbene in EGFR-mutation-positive non-small cell lung cancer (NSCLC). Int J Biol Sci. (2019) 15:2607–14. doi: 10.7150/ijbs.32889, PMID: 31754333 PMC6854375

[19] Hojo Y Kishi S Mori S Fujiwara-Tani R Sasaki T Fujii K. Sunitinib and pterostilbene combination treatment exerts antitumor effects in gastric cancer via suppression of PDZD8. Int J Mol Sci. (2022) 23:4002. doi: 10.3390/ijms23074002, PMID: 35409367 PMC8999764

[20] Obrador E Salvador-Palmer R Jihad-Jebbar A López-Blanch R Dellinger TH Dellinger RW. Pterostilbene in cancer therapy. Antioxidants (Basel). (2021) 10:492. doi: 10.3390/antiox10030492, PMID: 33801098 PMC8004113

[21] Lin WS Leland JV Ho CT Pan MH. Occurrence, bioavailability, anti-inflammatory, and anticancer effects of pterostilbene. J Agric Food Chem. (2020) 68:12788–99. doi: 10.1021/acs.jafc.9b07860, PMID: 32064876

[22] He P Li Y Hu J Deng B Tan Z Chen Y. Pterostilbene suppresses gastric cancer proliferation and metastasis by inhibiting oncogenic JAK2/STAT3 signaling: *In vitro* and *in vivo* therapeutic intervention. Phytomedicine. (2024) 128:155316. doi: 10.1016/j.phymed.2023.155316, PMID: 38518635

[23] Wang Y Zhang Q Chen Y Liang C-L Liu H Qiu F. Antitumor effects of immunity-enhancing traditional Chinese medicine. Biomedicine Pharmacother. (2020) 121:109570. doi: 10.1016/j.biopha.2019.109570, PMID: 31710893

[24] Wang XR Jiang ZB Xu C Meng WY Liu P Zhang YZ. Andrographolide suppresses non-small-cell lung cancer progression through induction of autophagy and antitumor immune response. Pharmacol Res. (2022) 179:106198. doi: 10.1016/j.phrs.2022.106198, PMID: 35367343

[25] Tan Y Zhu Q Yang M Yang F Zeng Q Jiang Z. Tetrandrine activates STING/TBK1/IRF3 pathway to potentiate anti-PD-1 immunotherapy efficacy in non-small cell lung cancer. Pharmacol Res. (2024) 207:107314. doi: 10.1016/j.phrs.2024.107314, PMID: 39059614

[26] Jiang ZB Huang JM Xie YJ Zhang YZ Chang C Lai HL. Evodiamine suppresses non-small cell lung cancer by elevating CD8(+) T cells and downregulating the MUC1-C/PD-L1 axis. J Exp Clin Cancer Res. (2020) 39:249. doi: 10.1186/s13046-020-01741-5, PMID: 33208183 PMC7677782

[27] Xie YJ Gao WN Wu QB Yao XJ Jiang ZB Wang YW. Chelidonine selectively inhibits the growth of gefitinib-resistant non-small cell lung cancer cells through the EGFR-AMPK pathway. Pharmacol Res. (2020) 159:104934. doi: 10.1016/j.phrs.2020.104934, PMID: 32464330

[28] Rosenberg M Azevedo NF Ivask A. Propidium iodide staining underestimates viability of adherent bacterial cells. Sci Rep. (2019) 9:6483. doi: 10.1038/s41598-019-42906-3, PMID: 31019274 PMC6482146

[29] Xu C Jiang ZB Shao L Zhao ZM Fan XX Sui X. β-Elemene enhances erlotinib sensitivity through induction of ferroptosis by upregulating lncRNA H19 in EGFR-mutant non-small cell lung cancer. Pharmacol Res. (2023) 191:106739. doi: 10.1016/j.phrs.2023.106739, PMID: 36948327

[30] Jiang ZB Xu C Wang W Zhang YZ Huang JM Xie YJ. Plumbagin suppresses non-small cell lung cancer progression through downregulating ARF1 and by elevating CD8(+) T cells. Pharmacol Res. (2021) 169:105656. doi: 10.1016/j.phrs.2021.105656, PMID: 33964470

[31] Jiang ZB Wang WJ Xu C Xie YJ Wang XR Zhang YZ. Luteolin and its derivative apigenin suppress the inducible PD-L1 expression to improve anti-tumor immunity in KRAS-mutant lung cancer. Cancer Lett. (2021) 515:36–48. doi: 10.1016/j.canlet.2021.05.019, PMID: 34052328

[32] Maeda T Hiraki M Jin C Rajabi H Tagde A Alam M. MUC1-C induces PD-L1 and immune evasion in triple-negative breast cancer. Cancer Res. (2018) 78:205–15. doi: 10.1158/0008-5472.CAN-17-1636, PMID: 29263152 PMC5754244

[33] C. National Research Council Committee for the Update of the Guide for the and A. Use of Laboratory, The National Academies Collection: Reports funded by National Institutes of Health, Guide for the Care and Use of Laboratory Animals. Washington (DC: National Academies Press (US) Copyright © 2011, National Academy of Sciences. (2011).

[34] Yang Z Chu B Tu Y Li L Chen D Huang S. Dual inhibitors of DNMT and HDAC remodels the immune microenvironment of colorectal cancer and enhances the efficacy of anti-PD-L1 therapy. Pharmacol Res. (2024) 206:107271. doi: 10.1016/j.phrs.2024.107271, PMID: 38906202

[35] Zhang YZ Lai HL Huang C Jiang ZB Yan HX Wang XR. Tanshinone IIA induces ER stress and JNK activation to inhibit tumor growth and enhance anti-PD-1 immunotherapy in non-small cell lung cancer. Phytomedicine. (2024) 128:155431. doi: 10.1016/j.phymed.2024.155431, PMID: 38537440

[36] Brix N Samaga D Belka C Zitzelsberger H Lauber K. Analysis of clonogenic growth *in vitro* . Nat Protoc. (2021) 16:4963–91. doi: 10.1038/s41596-021-00615-0, PMID: 34697469

[37] Nakamura H Takada K. Reactive oxygen species in cancer: Current findings and future directions. Cancer Sci. (2021) 112:3945–52. doi: 10.1111/cas.15068, PMID: 34286881 PMC8486193

[38] Gao H Liu Z Xu W Wang Q Zhang C Ding Y. Pterostilbene promotes mitochondrial apoptosis and inhibits proliferation in glioma cells. Sci Rep. (2021) 11:6381. doi: 10.1038/s41598-021-85908-w, PMID: 33737656 PMC7973728

[39] Malli Cetinbas N Monnell T Soomer-James J Shaw P Lancaster K Catcott KC. Tumor cell-directed STING agonist antibody-drug conjugates induce type III interferons and anti-tumor innate immune responses. Nat Commun. (2024) 15:5842. doi: 10.1038/s41467-024-49932-4, PMID: 38992037 PMC11239908

[40] Shinde O Li P. Chapter One - The molecular mechanism of dsDNA sensing through the cGAS-STING pathway. : F.W Alt K.M Murphy (Eds.) Adv Immunol Acad Press. (2024) pp:1–21. doi: 10.1016/bs.ai.2024.02.003, PMID: 38866436

[41] Kobritz M Borjas T Patel V Coppa G Aziz M Wang P. H151, A small molecule inhibitor of sting as A novel therapeutic in intestinal ischemia-reperfusion injury. Shock. (2022) 58:241–50. doi: 10.1097/SHK.0000000000001968, PMID: 35959789 PMC9489661

[42] Hartmann W Blankenhaus B Brunn M-L Meiners J Breloer M. Elucidating different pattern of immunoregulation in BALB/c and C57BL/6 mice and their F1 progeny. Sci Rep. (2021) 11:1536. doi: 10.1038/s41598-020-79477-7, PMID: 33452272 PMC7810711

[43] Mohd Kamal K Ghazali AR Ab Mutalib NS Abu N Chua EW Masre SF. The role of DNA methylation and DNA methyltransferases (DNMTs) as potential biomarker and therapeutic target in non-small cell lung cancer (NSCLC). Heliyon. (2024) 10:e38663. doi: 10.1016/j.heliyon.2024.e38663, PMID: 39403460 PMC11472108

[44] Lanng KRB Lauridsen EL Jakobsen MR. The balance of STING signaling orchestrates immunity in cancer. Nat Immunol. (2024) 25:1144–57. doi: 10.1038/s41590-024-01872-3, PMID: 38918609

[45] Leiter A Veluswamy RR Wisnivesky JP. The global burden of lung cancer: current status and future trends. Nat Rev Clin Oncol. (2023) 20:624–39. doi: 10.1038/s41571-023-00798-3, PMID: 37479810

[46] Hendriks LEL Remon J Faivre-Finn C Garassino MC Heymach JV Kerr KM. Non-small-cell lung cancer. Nat Rev Dis Primers. (2024) 10:71. doi: 10.1038/s41572-024-00551-9, PMID: 39327441

[47] Araghi M Mannani R Heidarnejad maleki A Hamidi A Rostami S Safa SH. Recent advances in non-small cell lung cancer targeted therapy; an update review. Cancer Cell Int. (2023) 23:162. doi: 10.1186/s12935-023-02990-y, PMID: 37568193 PMC10416536

[48] Liu B Zhou H Tan L Siu KTH Guan X-Y. Exploring treatment options in cancer: Tumor treatment strategies. Signal Transduction Targeted Ther. (2024) 9:175. doi: 10.1038/s41392-024-01856-7, PMID: 39013849 PMC11252281

[49] Lustberg MB Kuderer NM Desai A Bergerot C Lyman GH. Mitigating long-term and delayed adverse events associated with cancer treatment: implications for survivorship. Nat Rev Clin Oncol. (2023) 20:527–42. doi: 10.1038/s41571-023-00776-9, PMID: 37231127 PMC10211308

[50] Miethke M Pieroni M Weber T Brönstrup M Hammann P Halby L. Towards the sustainable discovery and development of new antibiotics. Nat Rev Chem. (2021) 5:726–49. doi: 10.1038/s41570-021-00313-1, PMID: 34426795 PMC8374425

[51] Yadav N Deshmukh R Mazumder R. A comprehensive review on the use of traditional Chinese medicine for cancer treatment. Pharmacol Res - Modern Chin Med. (2024) 11:100423. doi: 10.1016/j.prmcm.2024.100423

[52] Liu P Tang W Xiang K Li G. Pterostilbene in the treatment of inflammatory and oncological diseases. Front Pharmacol. (2023) 14:1323377. doi: 10.3389/fphar.2023.1323377, PMID: 38259272 PMC10800393

[53] Liu Y You Y Lu J Chen X Yang Z. Recent advances in synthesis, bioactivity, and pharmacokinetics of pterostilbene, an important analog of resveratrol. Molecules. (2020) 25:5166. doi: 10.3390/molecules25215166, PMID: 33171952 PMC7664215

[54] Hsu YH Chen SY Wang SY Lin JA Yen GC. Pterostilbene enhances cytotoxicity and chemosensitivity in human pancreatic cancer cells. Biomolecules. (2020) 10:709. doi: 10.3390/biom10050709, PMID: 32375296 PMC7281188

[55] Wang Z Wang T Chen X Cheng J Wang L. Pterostilbene regulates cell proliferation and apoptosis in non-small-cell lung cancer via targeting COX-2. Biotechnol Appl Biochem. (2023) 70:106–19. doi: 10.1002/bab.2332, PMID: 35231150

[56] Byun WS Bae ES Kim WK Lee SK. Antitumor activity of rutaecarpine in human colorectal cancer cells by suppression of Wnt/β-catenin signaling. J Nat Prod. (2022) 85:1407–18. doi: 10.1021/acs.jnatprod.2c00224, PMID: 35544614

[57] Liu YQ Zhou GB. Promising anticancer activities and mechanisms of action of active compounds from the medicinal herb Centipeda minima (L. ) A Braun Asch Phytomedicine. (2022) 106:154397. doi: 10.1016/j.phymed.2022.154397, PMID: 36084403

[58] You M Xie Z Zhang N Zhang Y Xiao D Liu S. Signaling pathways in cancer metabolism: mechanisms and therapeutic targets. Signal Transduction Targeted Ther. (2023) 8:196. doi: 10.1038/s41392-023-01442-3, PMID: 37164974 PMC10172373

[59] Li X Gao J Wu C Wang C Zhang R He J. Precise modulation and use of reactive oxygen species for immunotherapy. Sci Adv. (2024) 10:eadl0479. doi: 10.1126/sciadv.adl0479, PMID: 38748805 PMC11095489

[60] Glorieux C Liu S Trachootham D Huang P. Targeting ROS in cancer: rationale and strategies. Nat Rev Drug Discov. (2024). 23:583–606. doi: 10.1038/s41573-024-00979-4, PMID: 38982305

[61] Sies H Belousov VV Chandel NS Davies MJ Jones DP Mann GE. Defining roles of specific reactive oxygen species (ROS) in cell biology and physiology. Nat Rev Mol Cell Biol. (2022) 23:499–515. doi: 10.1038/s41580-022-00456-z, PMID: 35190722

[62] Perillo B Di Donato M Pezone A Di Zazzo E Giovannelli P Galasso G. ROS in cancer therapy: the bright side of the moon. Exp Mol Med. (2020) 52:192–203. doi: 10.1038/s12276-020-0384-2, PMID: 32060354 PMC7062874

[63] Decout A Katz JD Venkatraman S Ablasser A. The cGAS–STING pathway as a therapeutic target in inflammatory diseases. Nat Rev Immunol. (2021) 21:548–69. doi: 10.1038/s41577-021-00524-z, PMID: 33833439 PMC8029610

[64] Di Giulio S Colicchia V Pastorino F Pedretti F Fabretti F Nicolis di Robilant V. A combination of PARP and CHK1 inhibitors efficiently antagonizes MYCN-driven tumors. Oncogene. (2021) 40:6143–52. doi: 10.1038/s41388-021-02003-0, PMID: 34508175 PMC8553625

[65] Binnewies M Roberts EW Kersten K Chan V Fearon DF Merad M. Understanding the tumor immune microenvironment (TIME) for effective therapy. Nat Med. (2018) 24:541–50. doi: 10.1038/s41591-018-0014-x, PMID: 29686425 PMC5998822

[66] Wang D-R Wu X-L Sun Y-L. Therapeutic targets and biomarkers of tumor immunotherapy: response versus non-response. Signal Transduction Targeted Ther. (2022) 7:331. doi: 10.1038/s41392-022-01136-2, PMID: 36123348 PMC9485144

[67] Zhang A Fan T Liu Y Yu G Li C Jiang Z. Regulatory T cells in immune checkpoint blockade antitumor therapy. Mol Cancer. (2024) 23:251. doi: 10.1186/s12943-024-02156-y, PMID: 39516941 PMC11545879

[68] Xia L Oyang L Lin J Tan S Han Y Wu N. The cancer metabolic reprogramming and immune response. Mol Cancer. (2021) 20:28. doi: 10.1186/s12943-021-01316-8, PMID: 33546704 PMC7863491

[69] Zhou Z Zheng J Lu Y Mai Z Lin Y Lin P. Optimizing CD8+ T cell-based immunotherapy via metabolic interventions: a comprehensive review of intrinsic and extrinsic modulators. Exp Hematol Oncol. (2024) 13:103. doi: 10.1186/s40164-024-00575-7, PMID: 39438986 PMC11495118

[70] Munoz LE Huang L Bommireddy R Sharma R Monterroza L Guin RN. Metformin reduces PD-L1 on tumor cells and enhances the anti-tumor immune response generated by vaccine immunotherapy. J Immunother Cancer. (2021) 9:e002614. doi: 10.1136/jitc-2021-002614, PMID: 34815353 PMC8611422

[71] Cha JH Yang WH Xia W Wei Y Chan LC Lim SO. Metformin promotes antitumor immunity via endoplasmic-reticulum-associated degradation of PD-L1. Mol Cell. (2018) 71:606–20.e7. doi: 10.1016/j.molcel.2018.07.030, PMID: 30118680 PMC6786495

[72] Park SH Lee J Yun HJ Kim SH Lee JH. Metformin suppresses both PD-L1 expression in cancer cells and cancer-induced PD-1 expression in immune cells to promote antitumor immunity. Ann Lab Med. (2024) 44:426–36. doi: 10.3343/alm.2023.0443, PMID: 38529546 PMC11169777

[73] Kapetanovic IM Muzzio M Huang Z Thompson TN McCormick DL. Pharmacokinetics, oral bioavailability, and metabolic profile of resveratrol and its dimethylether analog, pterostilbene, in rats. Cancer Chemother Pharmacol. (2011) 68:593–601. doi: 10.1007/s00280-010-1525-4, PMID: 21116625 PMC3090701

[74] Dutta BJ Rakshe PS Maurya N Chib S Singh S. Unlocking the therapeutic potential of natural stilbene: Exploring pterostilbene as a powerful ally against aging and cognitive decline. Ageing Res Rev. (2023) 92:102125. doi: 10.1016/j.arr.2023.102125, PMID: 37979699

[75] Li J Li H Ye P Ou A Liu M Huang S. Stability and efficacy of pterostilbene nanoliposomes in cosmetic applications: A comprehensive study. J Dermatologic Sci Cosmetic Technol. (2024) 1:100056. doi: 10.1016/j.jdsct.2024.100056

[76] Memon D Schoenfeld AJ Ye D Fromm G Rizvi H Zhang X. Clinical and molecular features of acquired resistance to immunotherapy in non-small cell lung cancer. Cancer Cell (2024) 42:209–24.e209. doi: 10.1016/j.ccell.2023.12.011, PMID: 38215748 PMC11249385

[77] Oser MG MacPherson D Oliver TG Sage J Park K-S. Genetically-engineered mouse models of small cell lung cancer: the next generation. Oncogene. (2024) 43:457–69. doi: 10.1038/s41388-023-02929-7, PMID: 38191672 PMC11180418

[78] Sharma P Hu-Lieskovan S Wargo JA Ribas A. Primary, adaptive, and acquired resistance to cancer immunotherapy. Cell. (2017) 168:707–23. doi: 10.1016/j.cell.2017.01.017, PMID: 28187290 PMC5391692

