# Supplementary figures and images for: Pterostilbene inhibits non-small cell lung cancer progression by activating the STING pathway and enhancing antitumor immune response
Source: Front Immunol. 2025 Oct 16;16:1622284. doi: 10.3389/fimmu.2025.1622284 (PMC12571870; doi:10.3389/fimmu.2025.1622284)

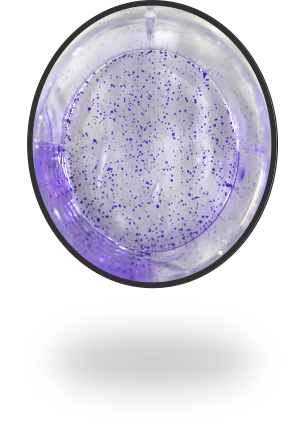

Supplement: Supplementary file 1 [file DataSheet1.zip › Figure/Figure 2/Figure 2A-A549-0.png]

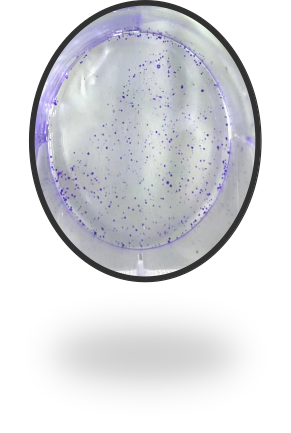

Supplement: Supplementary file 1 [file DataSheet1.zip › Figure/Figure 2/Figure 2A-A549-CDDP.png]

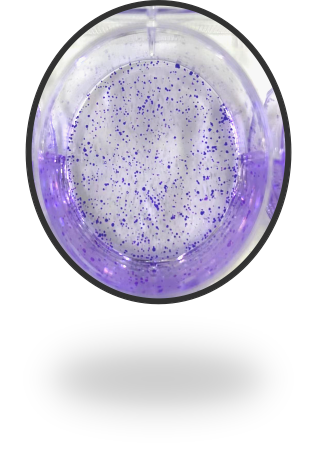

Supplement: Supplementary file 1 [file DataSheet1.zip › Figure/Figure 2/Figure 2A-A549-PTE 10μM.png]

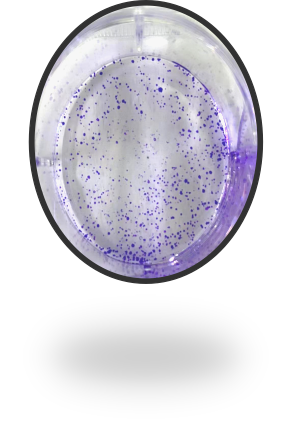

Supplement: Supplementary file 1 [file DataSheet1.zip › Figure/Figure 2/Figure 2A-A549-PTE 20μM.png]

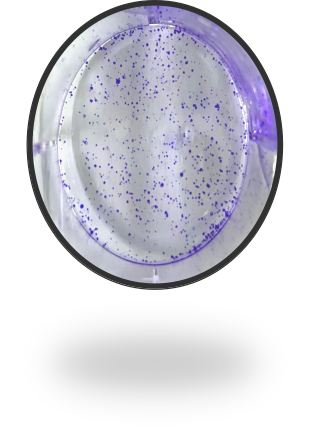

Supplement: Supplementary file 1 [file DataSheet1.zip › Figure/Figure 2/Figure 2A-A549-PTE 30μM.png]

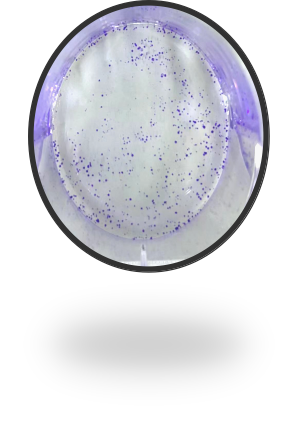

Supplement: Supplementary file 1 [file DataSheet1.zip › Figure/Figure 2/Figure 2A-A549-PTE 40μM.png]

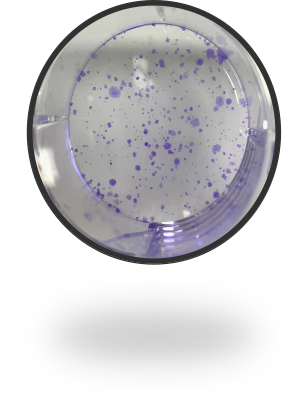

Supplement: Supplementary file 1 [file DataSheet1.zip › Figure/Figure 2/Figure 2A-H358-0.png]

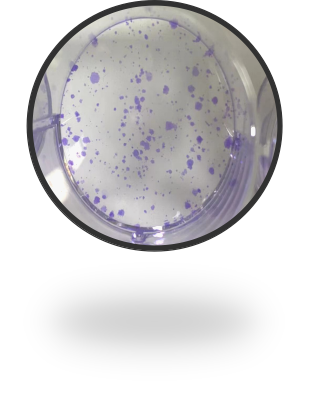

Supplement: Supplementary file 1 [file DataSheet1.zip › Figure/Figure 2/Figure 2A-H358-PTE 10μM.png]

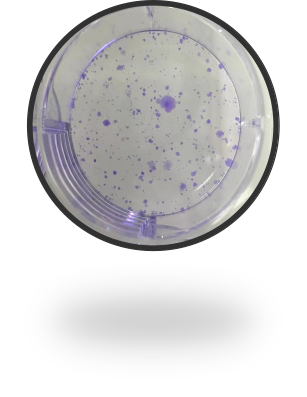

Supplement: Supplementary file 1 [file DataSheet1.zip › Figure/Figure 2/Figure 2A-H358-PTE 20μM.png]

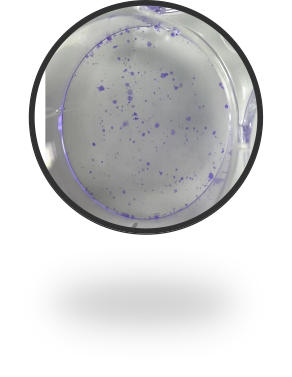

Supplement: Supplementary file 1 [file DataSheet1.zip › Figure/Figure 2/Figure 2A-H358-PTE 30μM.png]

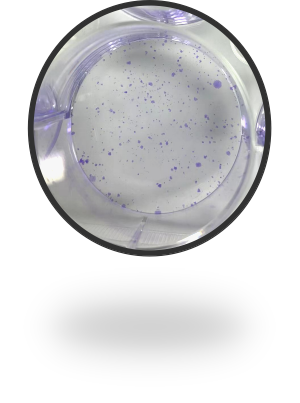

Supplement: Supplementary file 1 [file DataSheet1.zip › Figure/Figure 2/Figure 2A-H358-PTE 40μM.png]

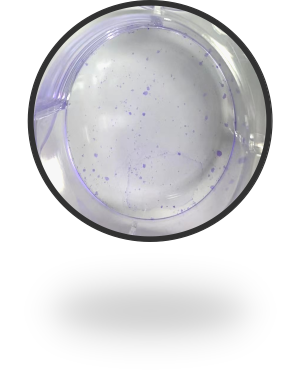

Supplement: Supplementary file 1 [file DataSheet1.zip › Figure/Figure 2/Figure 2A-H358-PTE CDDP.png]

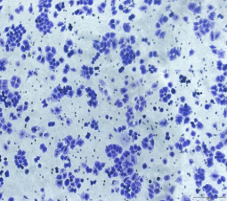

Supplement: Supplementary file 1 [file DataSheet1.zip › Figure/Figure 2/Figure 2C-A549-0.png]

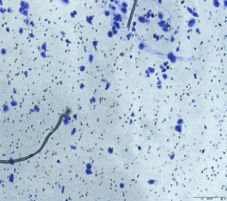

Supplement: Supplementary file 1 [file DataSheet1.zip › Figure/Figure 2/Figure 2C-A549-CDDP.png]

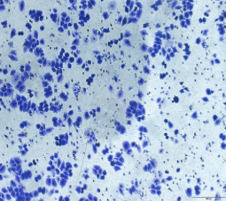

Supplement: Supplementary file 1 [file DataSheet1.zip › Figure/Figure 2/Figure 2C-A549-PTE 10μM.png]

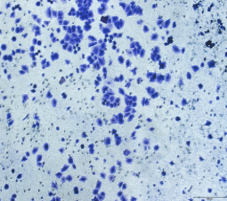

Supplement: Supplementary file 1 [file DataSheet1.zip › Figure/Figure 2/Figure 2C-A549-PTE 20μM.png]

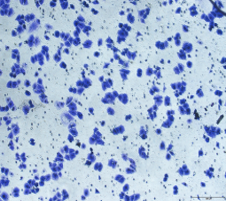

Supplement: Supplementary file 1 [file DataSheet1.zip › Figure/Figure 2/Figure 2C-A549-PTE 30μM.png]

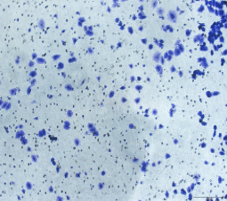

Supplement: Supplementary file 1 [file DataSheet1.zip › Figure/Figure 2/Figure 2C-A549-PTE 40μM.png]

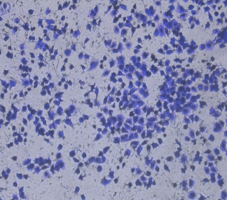

Supplement: Supplementary file 1 [file DataSheet1.zip › Figure/Figure 2/Figure 2C-H358-0.png]

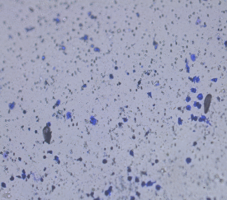

Supplement: Supplementary file 1 [file DataSheet1.zip › Figure/Figure 2/Figure 2C-H358-CDDP.png]

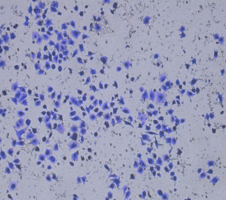

Supplement: Supplementary file 1 [file DataSheet1.zip › Figure/Figure 2/Figure 2C-H358-PTE 10μM.png]

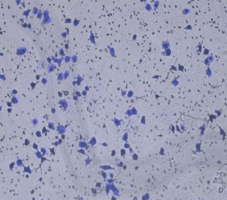

Supplement: Supplementary file 1 [file DataSheet1.zip › Figure/Figure 2/Figure 2C-H358-PTE 20μM.png]

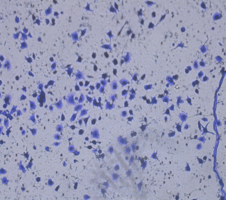

Supplement: Supplementary file 1 [file DataSheet1.zip › Figure/Figure 2/Figure 2C-H358-PTE 30μM.png]

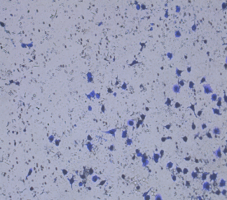

Supplement: Supplementary file 1 [file DataSheet1.zip › Figure/Figure 2/Figure 2C-H358-PTE 40μM.png]

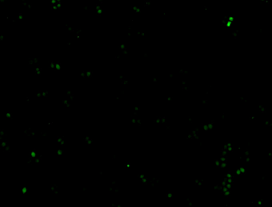

Supplement: Supplementary file 1 [file DataSheet1.zip › Figure/Figure 4/Figure 4C-A549-0.png]

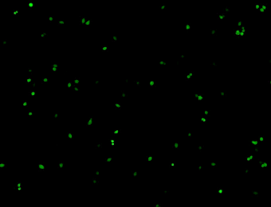

Supplement: Supplementary file 1 [file DataSheet1.zip › Figure/Figure 4/Figure 4C-A549-10 UM.png]

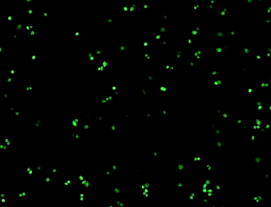

Supplement: Supplementary file 1 [file DataSheet1.zip › Figure/Figure 4/Figure 4C-A549-20 UM.png]

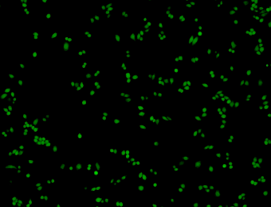

Supplement: Supplementary file 1 [file DataSheet1.zip › Figure/Figure 4/Figure 4C-A549-30 UM.png]

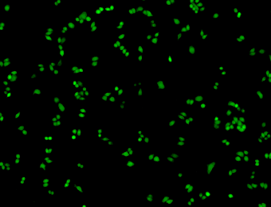

Supplement: Supplementary file 1 [file DataSheet1.zip › Figure/Figure 4/Figure 4C-A549-40 UM.png]

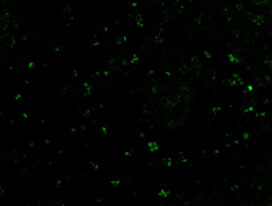

Supplement: Supplementary file 1 [file DataSheet1.zip › Figure/Figure 4/Figure 4C-H358-0.png]

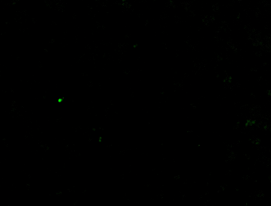

Supplement: Supplementary file 1 [file DataSheet1.zip › Figure/Figure 4/Figure 4C-H358-10 UM.png]

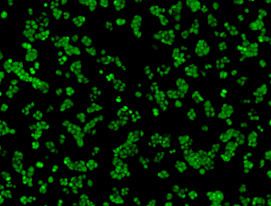

Supplement: Supplementary file 1 [file DataSheet1.zip › Figure/Figure 4/Figure 4C-H358-20 UM.png]

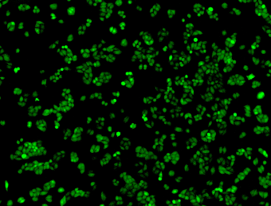

Supplement: Supplementary file 1 [file DataSheet1.zip › Figure/Figure 4/Figure 4C-H358-30 UM.png]

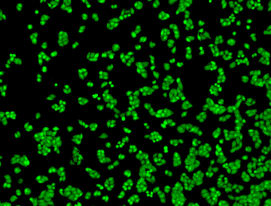

Supplement: Supplementary file 1 [file DataSheet1.zip › Figure/Figure 4/Figure 4C-H358-40 UM.png]

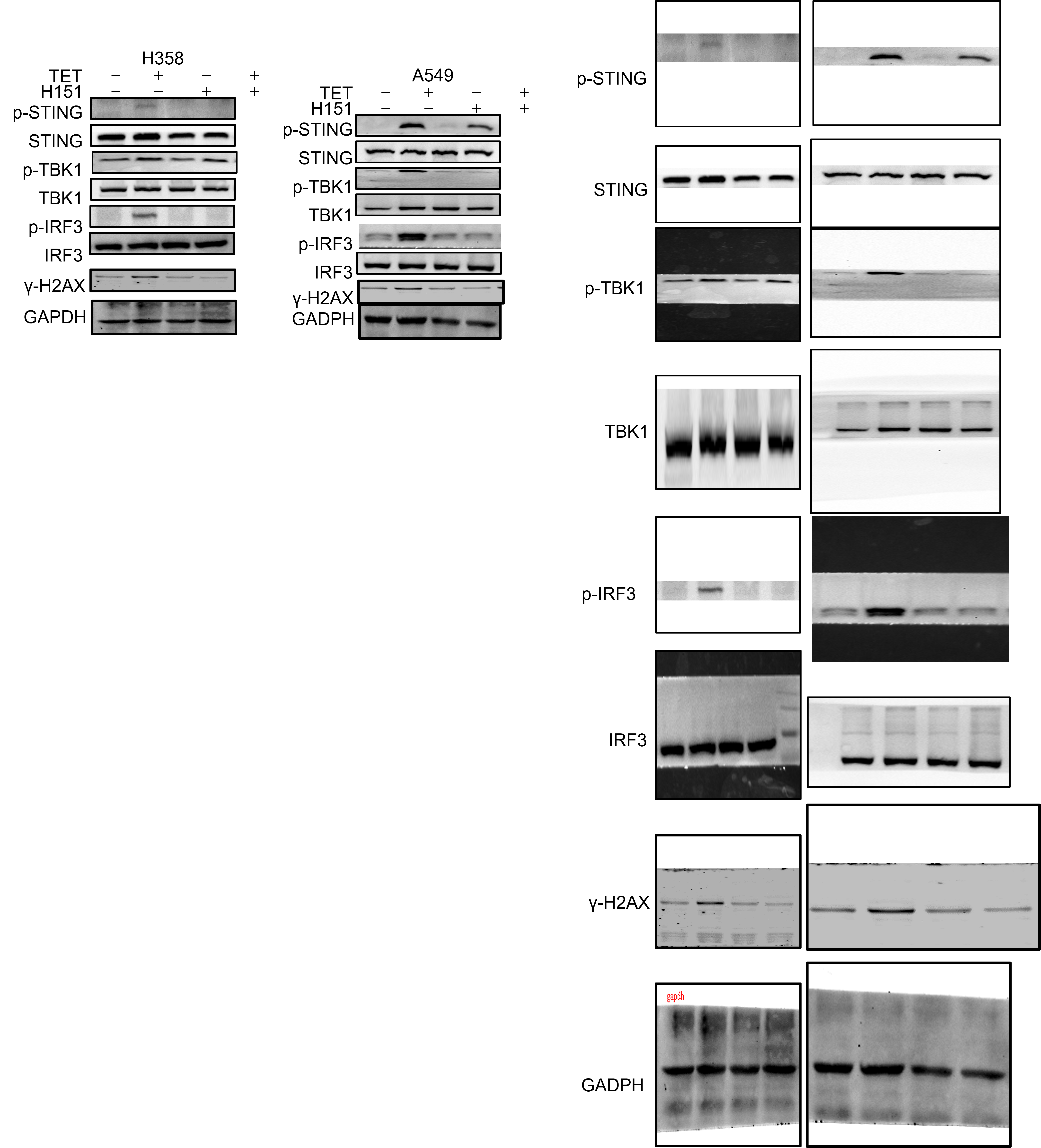

Supplement: Supplementary file 1 [file DataSheet1.zip › Figure/Figure 5/图片24.png]

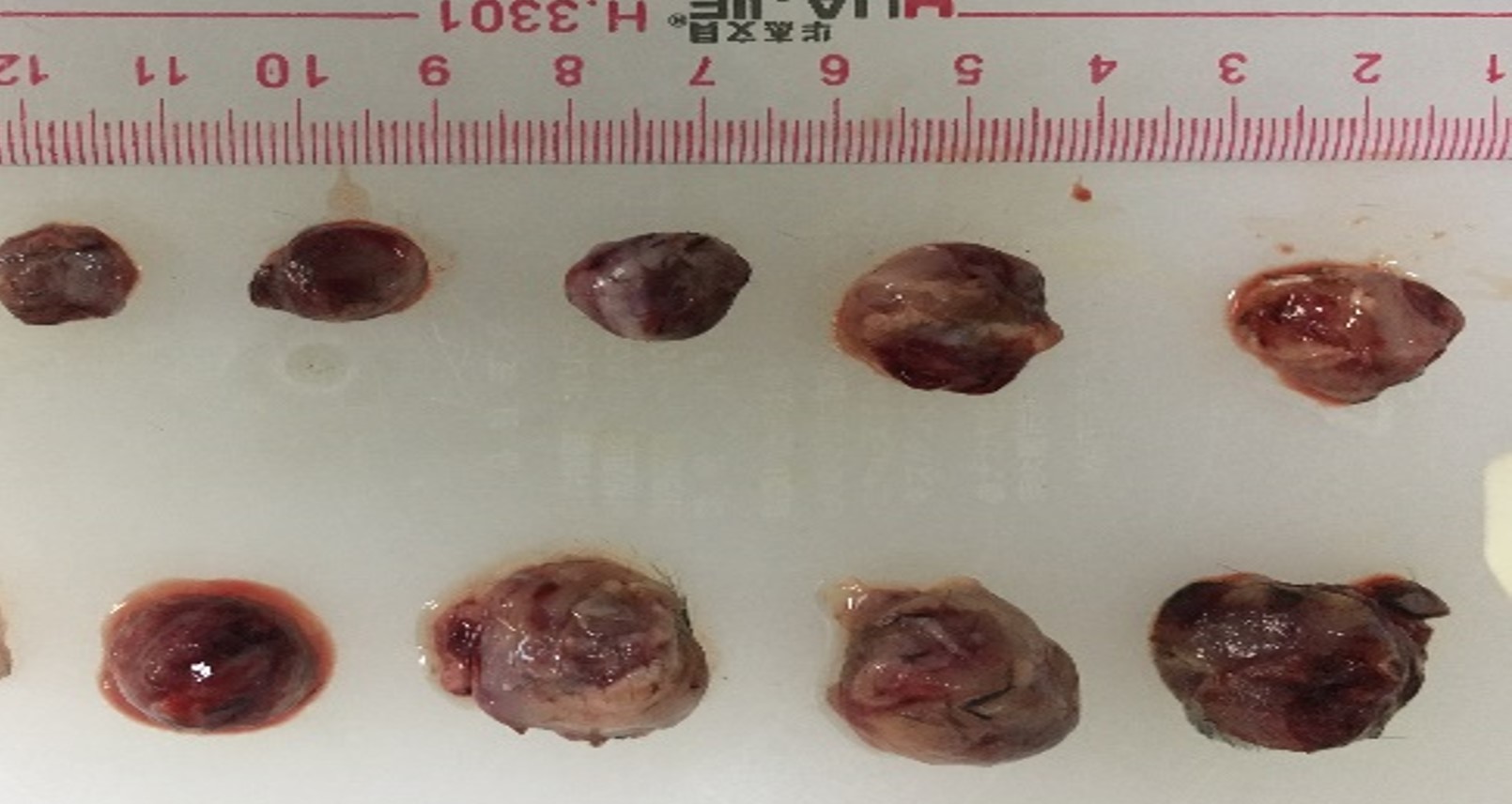

Supplement: Supplementary file 1 [file DataSheet1.zip › Figure/Figure 6/Figure 5D.jpg]

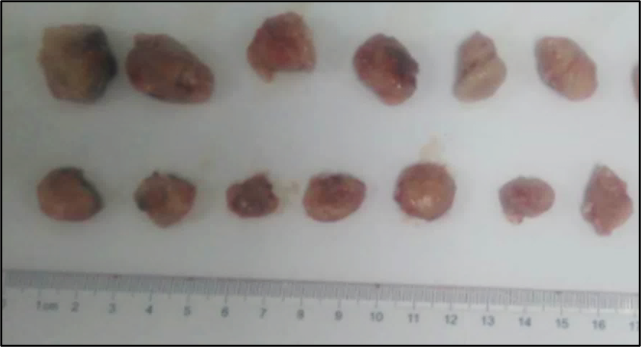

Supplement: Supplementary file 1 [file DataSheet1.zip › Figure/Figure 7/Figure 7c.png]

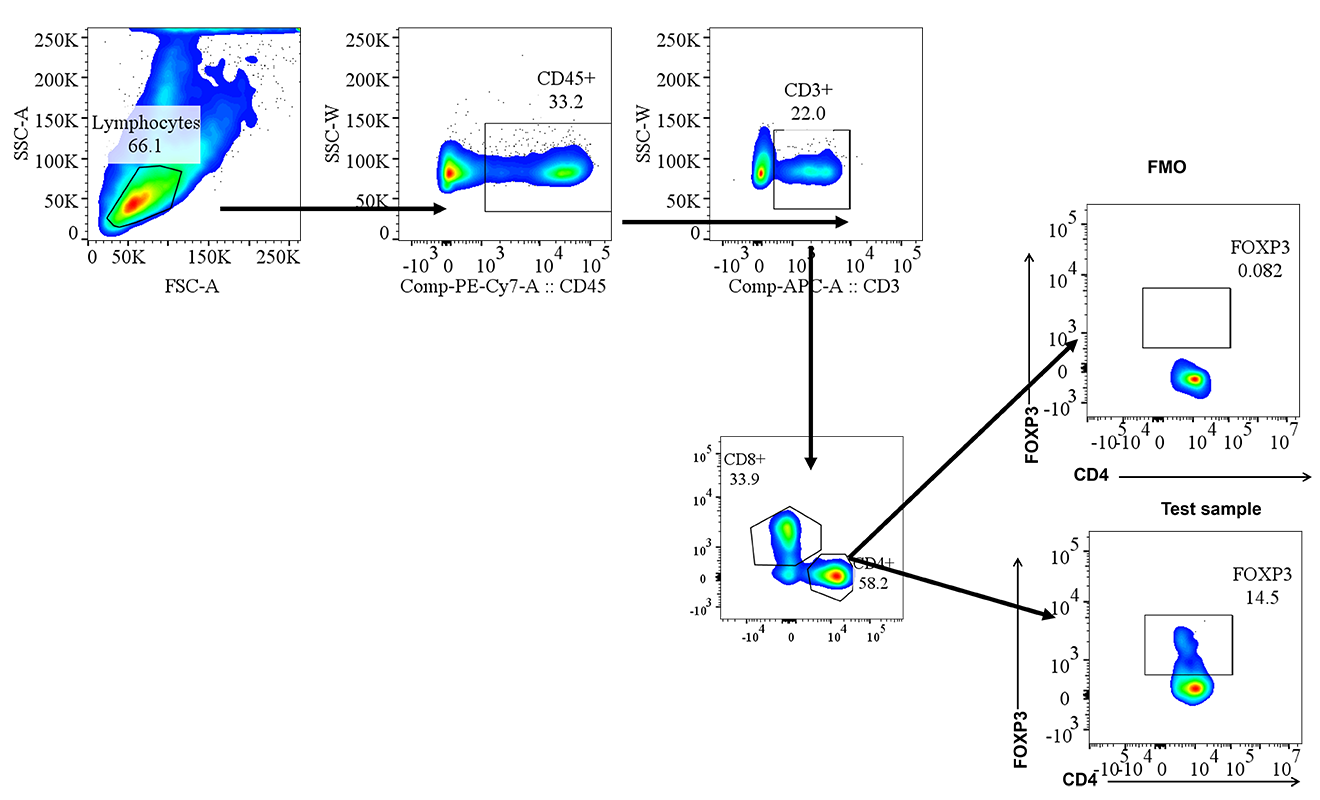

Supplement: Supplementary file 9 [file Image1.tif]

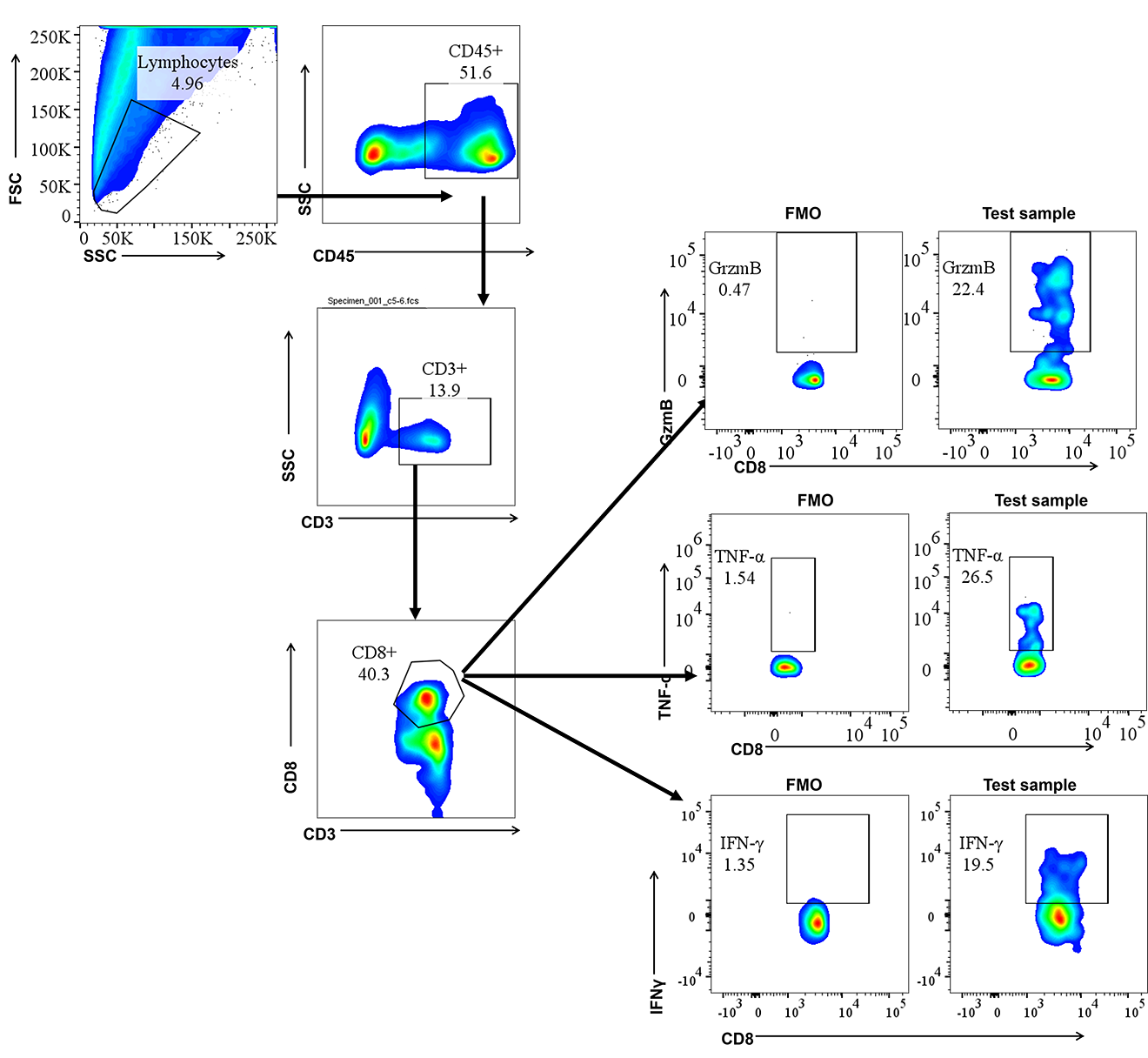

Supplement: Supplementary file 10 [file Image2.tif]
